# Acceptability of Digital Mental Health Interventions for Depression and Anxiety: Systematic Review

**DOI:** 10.2196/52609

**Published:** 2024-10-28

**Authors:** Carrie K Y Lau, Anthony Saad, Bettina Camara, Dia Rahman, Blanca Bolea-Alamanac

**Affiliations:** 1 Department of Psychiatry Women's College Hospital Toronto, ON Canada; 2 Factor-Inwentash Faculty of Social Work University of Toronto Toronto, ON Canada; 3 Department of Immunology Faculty of Medicine University of Toronto Toronto, ON Canada; 4 Department of Psychiatry University of Toronto Toronto, ON Canada

**Keywords:** acceptability, mental health, depression, anxiety, mobile app, internet, mobile phone, mobile health, mHealth, digital health

## Abstract

**Background:**

Depression and anxiety disorders are common, and treatment often includes psychological interventions. Digital health interventions, delivered through technologies such as web-based programs and mobile apps, are increasingly used in mental health treatment. Acceptability, the extent to which an intervention is viewed positively, has been identified as contributing to patient adherence and engagement with digital health interventions. Acceptability, therefore, impacts the benefit derived from using digital health interventions in treatment. Understanding the acceptability of digital mental health interventions among patients with depression or anxiety disorders is essential to maximize the effectiveness of their treatment.

**Objective:**

This review investigated the acceptability of technology-based interventions among patients with depression or anxiety disorders.

**Methods:**

A systematic review was performed based on PRISMA (Preferred Reporting Items for Systematic Reviews and Meta-Analyses) and PROSPERO (International Prospective Register of Systematic Reviews) guidelines. We searched PubMed, Web of Science, and Ovid in May 2022. Studies were included if they evaluated digital interventions for the treatment of depression or anxiety disorders and investigated their acceptability among adult patients. Studies were excluded if they targeted only specific populations (eg, those with specific physical health conditions), investigated acceptability in healthy individuals or patients under the age of 18 years, involved no direct interaction between patients and technologies, used technology only as a platform for traditional care (eg, videoconferencing), had patients using technologies only in clinical or laboratory settings, or involved virtual reality technologies. Acceptability outcome data were narratively synthesized by the direction of acceptability using vote counting. Included studies were evaluated using levels of evidence from the Oxford Centre for Evidence-Based Medicine. The risk of bias was assessed using a tool designed for this review and GRADE (Grading of Recommendations, Assessment, Development, and Evaluation).

**Results:**

A total of 143 articles met the inclusion criteria, comprising 67 (47%) articles on interventions for depression, 65 (45%) articles on interventions for anxiety disorders, and 11 (8%) articles on interventions for both. Overall, 90 (63%) were randomized controlled trials, 50 (35%) were other quantitative studies, and 3 (2%) were qualitative studies. Interventions used web-based programs, mobile apps, and computer programs. Cognitive behavioral therapy was the basis of 71% (102/143) of the interventions. Digital mental health interventions were generally acceptable among patients with depression or anxiety disorders, with 88% (126/143) indicating positive acceptability, 8% (11/143) mixed results, and 4% (6/143) insufficient information to categorize the direction of acceptability. The available research evidence was of moderate quality.

**Conclusions:**

Digital mental health interventions seem to be acceptable to patients with depression or anxiety disorders. Consistent use of validated measures for acceptability would enhance the quality of evidence. Careful design of acceptability as an evaluation outcome can further improve the quality of evidence and reduce the risk of bias.

**Trial Registration:**

Open Science Framework Y7MJ4; https://doi.org/10.17605/OSF.IO/SPR8M

## Introduction

### Background

Depression and anxiety disorders are common [1] and have high and increasing societal costs [2,3]. Patients with depression or anxiety disorders can benefit from psychological treatments but face a variety of barriers to accessing traditional mental health services.

Digital health interventions include those delivered with technologies such as web and mobile apps. They have garnered optimism for their potential to lower barriers to accessing mental health services [4,5]. Digital mental health interventions can facilitate access to services by increasing convenience for patients and reducing demands on their time, eliminating geographic limitations, reducing costs, and increasing the capacity of health systems [6].

The use of digital mental health interventions increased during the COVID-19 pandemic [7,8]. Their expanded reach occurred amid public health measures that limited in-person mental health treatment and predictions of a long tail of mental health repercussions [9]. Digital mental health interventions are now commonly available to patients alongside other mental health services, as an adjunct to in-person or synchronous video-based mental health treatment, or as a stopgap for long waitlists [10,11].

However, uncertainty exists around patient adherence to digital mental health interventions. Significant variations in adherence levels, ranging from low to high, have been noted among digital interventions [12]. Issues with nonadherence cast some doubt on whether the potential benefits of digital mental health interventions can be realized. Related issues have been found with user engagement (initiation and continuation of use of a digital health intervention) [13] among digital mental health interventions.

Acceptability, the degree to which individuals view a treatment or potential treatment positively, has been identified as predicting adherence and user engagement [14], and ultimately the intervention’s effectiveness [15]. Acceptability among patients is therefore critical to realizing the potential of a digital mental health intervention.

Research into acceptability appears hampered by its varying conceptualizations. Sekhon and colleagues [16] propose that acceptability is the patient’s perception of the treatment as appropriate, arising from their cognitive and affective reactions. They also provide a theoretical framework for acceptability with 7 components: affective attitude, burden, perceived effectiveness, ethicality, intervention coherence, opportunity costs, and self-efficacy [16]. Furthermore, Sekhon and colleagues [16] also recommend measuring acceptability at different time points during the intervention.

By contrast, Proctor and colleagues [17] define acceptability as the patient’s perception of the intervention as satisfactory or tolerable. Zauszniewski [18] describes acceptability as patients’ belief that the intervention is logical and suitable to them. They also describe 3 main components of acceptability, such as the acceptability of an intervention’s delivery method, acceptability of the content of an intervention, and acceptability of the provider of the intervention, although they acknowledge that acceptability is often assessed only as a whole [18]. In contrast, the technology acceptance model proposed by Davis [19], regarding attitudes toward technology and its use more generally, presents acceptability as hinging upon users’ perceptions of ease in using the technology and the technology’s usefulness.

Conflict between the constructs of acceptability and satisfaction has also been identified, with recommendations that acceptability be understood as distinct from satisfaction [16,17]. However, studies continue to operationalize acceptability as a patient’s satisfaction with a treatment, indicating continued resonance with the construction of satisfaction as a representation of acceptability, or at least a lack of consensus.

These examples demonstrate the variation with which acceptability has been understood and investigated in evaluations of digital mental health interventions thus far. Despite past recommendations for improvement (eg, Sekhon and colleagues [16]), evaluations have not yet converged on a particular approach to patient acceptability.

Acceptability has implications for user engagement and adherence [14]. Acceptability can impact the benefit that a patient can derive from an intervention and, in turn, compromise or enhance the individual’s investment into digital mental health interventions. It is of particular importance to investigate the acceptability of digital interventions for patients with depression and anxiety disorders, given the prevalence of these diagnoses and the frequency with which clinicians are required to identify appropriate psychosocial interventions [1].

### Aims and Objectives

This review summarizes the acceptability of digital mental health interventions for depression and anxiety disorders among adult patients. The research question we investigated was the following: “How acceptable to adult patients with depression or anxiety are digital mental health interventions (such as mobile apps) used in psychiatric care, and what is the evidence for their acceptability?” We accomplished this by identifying evaluation studies of digital mental health interventions where acceptability is one of the outcome measures, reviewing the quality of the available evidence for acceptability, and summarizing the acceptability of digital mental health interventions to patients with depression and anxiety disorders.

## Methods

### Overview

We started with Sekhon and colleagues’ [16] theoretical framework for acceptability, which identified in acceptability the component constructs of affective attitude, burden, perceived effectiveness, ethicality, intervention coherence, opportunity costs, and self-efficacy. We selected this theoretical framework because these component constructs can readily be identified in research studies having acceptability as an outcome measure. Where a study had an outcome measure that assessed one or more of these component constructs, whether or not the same terminology was used, the measure was considered relevant to our study.

In addition, the abovementioned theoretical framework for acceptability recognizes different temporal assessments of acceptability, such as prospective acceptability (before the intervention), concurrent acceptability (during the intervention), and retrospective acceptability (following the intervention) [16]. Each temporal assessment of acceptability is understood to achieve a different purpose and to provide different information [16]. For the purposes of this review, we included studies that assessed acceptability at any of these multiple time points but took note of the time points selected by the researchers for further analysis and discussion.

Sekhon and colleagues [16] suggest that satisfaction and acceptability are constructs that are often confounded but should be differentiated, referencing the fact that satisfaction can only be retrospectively assessed, while acceptability can be assessed at all time points. For this study, satisfaction is considered an aspect of retrospective acceptability. We therefore took into account measures of satisfaction as relevant to our review, despite satisfaction not being a part of Sekhon and colleagues’ [16] theoretical framework.

Other constructs not named within Sekhon and colleagues’ [16] theoretical framework of acceptability and included in this review are credibility, expectancy, and usability. For this review, studies evaluating credibility, expectancy, and usability were eligible for potential inclusion as long as the measures assess one or more component constructs of acceptability. Studies identified by their authors as collecting feedback, experiences, attitudes, subjective appraisal, and subjective benefit were similarly eligible for inclusion if they provided information on one or more component constructs of acceptability.

### Inclusion and Exclusion Criteria

Studies were included if (1) they investigated digital mental health interventions that monitor or treat depression or anxiety disorders; (2) they evaluated internet-based, mobile, or computer-based mental health interventions, and could involve the use of such technologies as mobile apps or web-based treatment modules; (3) their participants were 18 years of age or older; and (4) they collected data on the acceptability of the intervention to their participants.

Excluded were studies that (1) focused on behavioral or lifestyle changes in healthy people; (2) focused on a physical health condition, a neurodevelopmental condition, or a mental health diagnosis other than depression or an anxiety disorder; (3) focused on a specific population such as military personnel, health care workers, students, or a target group representing only one component of a larger population; (4) focused on family members or caregivers; (5) evaluated technologies that act as a conduit to traditional care but add no potential therapeutic value, such as videoconferencing platforms used for remote psychotherapy; (6) evaluated interventions that used virtual reality technology; (7) did not have participants interacting directly with the technology for therapeutic purposes (eg, sensors used by clinicians to collect data without offering therapeutic feedback to participants); (8) had participants <18 years of age; (9) had participants using the technology exclusively in clinical or laboratory settings; or (10) were not completed at the time of searching (eg, study protocols captured in database searches).

### Database Review

The review was conducted in 3 locations, PubMed, Web of Science (all databases in their Core Collection), and Ovid (all databases in their Health Sciences, Life Sciences, and Social Sciences categories). Search terms comprising both free text or natural language and Medical Subject Headings (MeSH) were applied in these searches (Multimedia Appendix 1). The searches were performed in May 2022 with no filters used to restrict the time period of the articles, and as a result, the oldest article in our search dated back to 1987. Reverse searches were also conducted by reviewing the references of relevant systematic reviews and meta-analyses, and by reviewing the references of included articles. Only articles with English abstracts were considered, but main texts could be in English, French, German, Italian, Spanish, or Chinese. Searches for gray literature using the same terms identified relevant published dissertations. PRISMA (Preferred Reporting Items for Systematic Reviews and Meta-Analyses) and PROSPERO (International Prospective Register of Systematic Reviews) [20] guided this review. The review was conducted using conventions for systematic reviews, screening first by title and abstract, followed by reviewing the complete article text. Furthermore, 2 reviewers (CKYL and one of AS, BC, or DR) independently assessed each title and abstract, and each complete text, to determine inclusion or exclusion. Disagreements in these assessments were resolved with further discussion (involving the reviewers and BB-A). The database software Covidence (Veritas Health Innovation Ltd) was used to organize and review the studies [21].

### Data Extraction and Synthesis

Data were captured from the included articles by the first author (CKYL) in a table format, checked by the last author (BB-A), and approved by all the authors. These data comprised the following: (1) depression or anxiety disorders needed for study eligibility, (2) details of the intervention (ie, mode of technology used, program name if one exists, and treatment paradigm), (3) details of the participant sample (ie, sample size, breakdown by group allocation, and gender), (4) details of acceptability-related outcomes (ie, outcome name, instrument used, and time point of data collection), and (5) results of acceptability-related outcomes. From these data, we noted considerable variation in instruments used to measure acceptability and infrequent use of validated instruments. Acceptability outcome data were therefore narratively synthesized by the direction of acceptability and not by its effect size. The narrative synthesis applied vote counting to the tabulated results, was conducted by the first author (CKYL), and was checked by the last author (BB-A).

### Levels of Evidence

Included studies were evaluated using the Oxford Centre for Evidence-Based Medicine–levels of evidence [22]. In this system, each study receives a designation based on a grading classification where 1 is the highest level (randomized controlled trials [RCTs] or systematic reviews of RCTs) and 5 is the lowest (mechanism-based reasoning).

Reported evidence for patient acceptability was further evaluated using an assessment tool designed for this review (Multimedia Appendix 2). The purpose of the assessment tool was to evaluate the quality of acceptability measurement and reporting. An assessment tool was developed because, to our knowledge, one did not already exist. Given the frequency (and variation) with which acceptability is reported as an evaluation outcome, the assessment tool allows summarization of our concerns with the current evidence. The tool emphasizes acquiring and providing sufficient information to evaluate, operationalize, and measure acceptability. Sekhon and colleagues’ [16] recommendation that acceptability be measured at multiple time points informed item 4 of the tool. The assessment tool produces a score ranging from 0 to 15, where scores from 0 to 5 indicate low quality of evidence for patient acceptability, scores 6-10 indicate moderate quality, and scores 11-15 indicate high quality. Assessments using this tool were performed by one author (CKYL) and reviewed by another (BB-A), with discrepancies identified and consensus achieved through discussion.

### Risk of Bias

Included studies were individually assessed on aspects of risk of bias using the assessment tool designed for this review (Multimedia Appendix 2). Specifically, the completeness of outcome data and the appropriateness of measurements (ie, selection of instruments) were determined using this assessment tool. These criteria were selected to assess each included study for their direct relevance to the single implementation outcome (ie, acceptability) that is the focus of this review. Preliminary assessments on these criteria indicated a significant risk of bias across the included studies, in terms of incomplete outcome data and low use of validated instruments.

Given these preliminary results suggesting a significant risk of bias, and this review’s focus on a single implementation outcome, risk of bias was assessed using GRADE (Grading of Recommendations, Assessment, Development and Evaluation) [23]. GRADE assesses the quality of evidence for risk of bias across a group of studies in relation to a particular outcome [23]. Across a body of evidence, the risk of bias is assessed as low, unclear, or high [23]. This evaluation was done independently by 2 authors (CKYL and BB-A), with discrepancy resolved with further discussion.

## Results

### Overview

**Figure 1:**
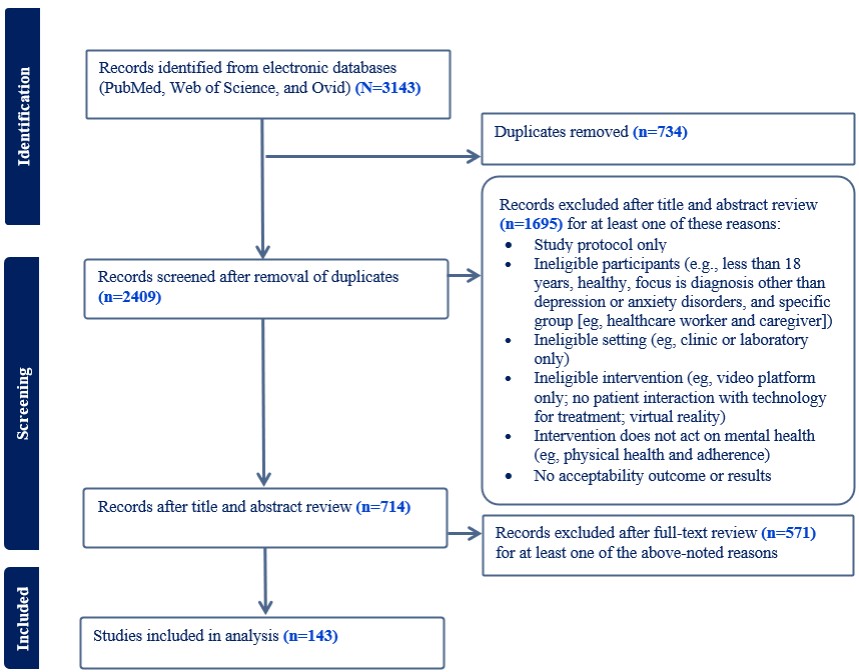
PRISMA (Preferred Reporting Items for Systematic Reviews and Meta-Analyses) flow diagram for each step of the screening process.

From the database review process, a total of 2409 articles were identified for screening. Of these articles, 2266 were excluded as they did not meet the inclusion criteria. Included in the review were 143 articles, investigating digital mental health interventions for depression (n=67, 47%), anxiety disorders (n=65, 45%), and a combination of depression and anxiety disorders (n=11, 8%; Figure 1 provides a flow diagram of the review).

In these articles, researchers most often reported on satisfaction as their acceptability-related construct of choice, with satisfaction investigated in 105 (73%) of the 143 included articles. Out of 143 articles, acceptability or acceptance was reported on in 78 (55%) articles, credibility in 26 (18%) articles, expectancy in 20 (14%) articles, and usability in 19 (13%) articles. There were also 21 (15%) articles using such terminology as feedback, experiences, attitudes, subjective appraisal, and subjective benefit.

For the evidence provided for acceptability and its component constructs, the vast majority, at 137 (96%) out of 143 articles, evaluated acceptability “globally” for each digital mental health intervention (ie, across features or components), and 20 (14%) articles assessed acceptability for specific features or components of each digital mental health intervention.

Referring to the component constructs of acceptability identified in Sekhon and colleagues’ [16] theoretical framework, each article investigated a subset of those component constructs. Specifically, out of 143 articles, 121 (85%) investigated affective attitude, 73 (51%) investigated perceived effectiveness, 37 (26%) investigated burden, and 35 (24%) investigated intervention coherence. The remaining component constructs were each investigated in 10% (15/143) or less of the articles (ie, opportunity cost: n=15, 10%; and self-efficacy: n=7, 5%). We did not find any articles that investigated ethicality.

Other constructs that were not explicit components of Sekhon and colleagues’ [16] theoretical framework for acceptability but were investigated in the reviewed articles included perceived quality (17/143, 12%) and acceptability of program tempo or length (10/143, 7%).

In terms of temporal assessments of acceptability or its component constructs, each article captured one or more of prospective acceptability (before the intervention began), concurrent acceptability (during the intervention), and retrospective acceptability (following the intervention), also set out in Sekhon and colleagues’ [16] theoretical framework. Prospective acceptability appeared in 42 (29%) of the 143 articles and concurrent acceptability in 9 (6%) articles. Retrospective acceptability was separated for this review into posttest (125/143, 87%) and follow-up (15/143, 10%). Of the total 143, only 43 (30%) articles contained acceptability assessments for more than 1 time point.

### Assessment of Quality

Using our assessment tool for quality of acceptability (Multimedia Appendix 2), articles investigating digital mental health interventions for depression had scores ranging from 2 (low quality of evidence) to 14 (high quality of evidence), out of a total possible 15. The mean score for the quality of evidence for acceptability was 7.3 (SD 2.0). From the information captured with this assessment tool, out of 143 articles, 74 (53%) used one or more validated instruments for acceptability or its component constructs, while 66 (47%) did not use any validated instruments for acceptability or its component constructs.

### Risk of Bias

The overall risk of bias was assessed as between high and unclear, considering the predominance of studies that lacked blinding (for RCTs), failed to adequately control confounding, had incomplete accounting of patients and outcome events (particularly losses to follow-up), and other limitations (eg, used measures that are not validated).

### Interventions for Depression

From the 67 articles addressing digital mental health interventions for depression, 40 (60%) articles involved web-based programs, 21 (31%) involved mobile apps, and 6 (9%) involved computer-based programs. In terms of treatment modality, 36 (54%) articles explored digital mental health interventions that drew from cognitive behavioral therapy (CBT). These apps or programs were often structured as multimedia modules, with varying levels of clinician or technician support and patient feedback.

The 67 articles investigating digital mental health interventions for depression were comprised of 41 (61%) RCTs, 23 (34%) non-RCT quantitative studies, and 3 (5%) qualitative studies. These studies are reviewed in Table S1 [24-65], Table S2 [66-89], and Table S3 [43,90-92], respectively, in Multimedia Appendix 3. The 64 quantitative studies (RCT and non-RCT) indicated overall positive ratings of acceptability among digital mental health interventions for depression. Among these 64 quantitative studies, we categorized 55 (86%) studies as indicating positive acceptability overall, 6 (9%) studies mixed acceptability, and 3 (5%) studies as having insufficient information to categorize the direction of acceptability. We considered results to be “mixed” if a study had more than 1 acceptability outcome measure and at least 1 positive and 1 negative result. These categorizations are presented in the “Summarized acceptability result” column within each table in Multimedia Appendix 3 [24-92].

The 3 qualitative studies (completed secondarily or subsequently to RCTs) had more mixed results. In 2014, Schneider and colleagues [90] reported that open-ended participant responses on satisfaction levels were more negative than positive, though satisfaction or dissatisfaction were reported as arising from matters related and unrelated to the interventions, such as individual time management choices made during the study. Comments specific to the web-based program were more positive than negative. Knowles and colleagues [91] reported in 2015 that they conducted interviews with participants using digital mental health interventions for depression (MoodGYM [e-hub Health] and Beating the Blues [Manage My Health], both web-based programs in CBT). Responses were coded as positive, negative, or ambivalent toward the digital mental health intervention, with the largest group being ambivalent (17/36 participants, 47%), then negative (10/36 participants, 28%), and then positive (9/36 participants, 25%). Also in 2015, Ly and colleagues [92] held semistructured interviews with participants using a mobile app informed by CBT and mindfulness techniques. Participants’ responses were divided, with 5 (42%) of 12 participants indicating an overall positive experience, 4 (33%) indicating a neutral experience, and 3 (25%) indicating a negative experience.

### Interventions for Anxiety Disorders

From the 65 articles addressing digital mental health interventions for anxiety, 52 (80%) articles involved web-based programs, 8 (12%) involved mobile apps, 3 (5%) involved computer-based programs, and 2 (3%) involved others. In terms of treatment modality, 58 (89%) articles explored digital mental health interventions that drew from CBT.

The 65 articles investigating digital mental health interventions for anxiety disorders were comprised of 43 (66%) RCTs and 22 (34%) non-RCT quantitative studies. None were qualitative studies. The studies are reviewed in Table S4 [93-135] and Table S5 [136-157] in Multimedia Appendix 3. In general, the results indicated positive ratings of acceptability. We categorized 59 (91%) studies as indicating positive acceptability overall, 3 (5%) studies mixed acceptability, and 3 (5%) studies as having insufficient information to categorize the direction of acceptability. We considered results to be “mixed” if a study had more than 1 acceptability outcome measure and at least 1 positive and 1 negative result. These categorizations are presented in the “Summarized acceptability result” column within each table in Multimedia Appendix 3 [93-157].

### Interventions for Depression and Anxiety Disorders Together

Among the 11 articles addressing digital mental health interventions for both depression and anxiety disorders, 8 (73%) articles involved web-based programs, 2 (18%) involved mobile apps, and 1 (9%) involved computer-based programs. In terms of treatment modality, 8 (73%) articles explored digital mental health interventions that drew from CBT.

The 11 articles investigating digital mental health interventions for both depression and anxiety disorders were comprised of 6 (55%) RCTs and 5 (45%) non-RCT quantitative studies (reviewed in Table S6 [158-163] and Table S7 [164-168] in Multimedia Appendix 3). None were qualitative studies. In general, the results indicated positive ratings of acceptability. We categorized all 11 studies as indicating positive acceptability overall, with none that indicated mixed results or insufficient information to categorize the direction of acceptability. These categorizations are presented in the “Summarized acceptability result” column within each table in Multimedia Appendix 3 [158-168].

## Discussion

### Principal Findings

Digital mental health interventions for depression and anxiety disorders were generally found to be acceptable to patients. Of the 143 included articles, 125 (87%) indicated positive acceptability, 12 (9%) had mixed results, and 6 had insufficient information (4%). These results arose out of evidence generated primarily from RCTs (90/143, 63%) and other quantitative studies (50/143, 35%), and largely investigations of web-based apps (100/143, 70%) and CBT-based content (102/143, 71%). These findings came primarily from views on a digital mental health intervention as a whole (137/143, 96%) rather than specific features, and centered on patient satisfaction (105/143, 73%) or Sekhon and colleagues’ [16] “affective attitude” (121/143, 85%) at posttest (125/143, 87%). We noted no substantial differences in the acceptability noted for digital mental health interventions for depression, anxiety, or depression and anxiety together.

Our findings suggest some receptiveness among those with depression or anxiety disorders to digital mental health interventions. If the overall positive experience of digital mental health interventions translates into patient adherence to and engagement with treatment, digital mental health interventions can improve patient access to care and expand the capacity of health systems. Since some patients appear open to digital mental health interventions, clinicians can explore patients’ views on these technologies in the course of treatment planning and respond accordingly. Researchers can further our understanding by investigating patient and intervention characteristics that predict or improve acceptance and deepen our understanding of acceptability beyond satisfaction or affective attitudes. Developers can recognize the potential in continuing to invest in digital mental health interventions and take steps to improve user experiences and strengthen acceptability.

The findings of positive acceptability toward digital mental health interventions must nevertheless be qualified by gaps in the existing research.

First, the assessment tool revealed that studies had a wide range in quality of acceptability-related evidence and were generally only of moderate quality. This finding suggests a need to increase standardization of acceptability assessments. We propose that our assessment tool be used as a checklist when planning evaluation research. The results outlined above indicate that there is an opportunity to use validated measures for acceptability, measure acceptability at different time points, and consider measures that address multiple acceptability components.

The second gap is the continued absence of a consensus definition for acceptability. As an example, Sekhon and colleagues [16] make clear that satisfaction ought to be treated as a construct separate from acceptability, but many studies continue to use satisfaction as an indicator of acceptability, or otherwise consider satisfaction alone when perhaps the construct of interest is acceptability. Similarly, Sekhon and colleagues [16] identified 7 component constructs of acceptability, but studies are generally designed to address only some of these components and vary by the components chosen. These findings suggest that there may not only be variation in how acceptability is investigated, but also variation in how acceptability is understood. It is necessary to establish a consensus on how acceptability should be defined, to increase the consistency and relevance of future acceptability research.

Third, some aspects of acceptability such as affective attitude and perceived effectiveness were more frequently investigated, while other aspects were far less likely to be explored, particularly ethicality, self-efficacy, and opportunity cost. Likely, this relates to some component constructs being considered more important than others, or the feasibility of investigating some component constructs over others. Investigating all components of acceptability is important to better understand how users relate to digital mental health interventions.

Finally, we noted a paucity of research on how acceptability changes over time, with most studies examining only retrospective acceptability. Evaluating acceptability as a variable that is continuous and changeable over time would enhance development processes for digital mental health interventions. It would allow us to identify the modifications that can be made to a technology to strengthen acceptability at different stages, to increase willingness to initiate use of a digital mental health intervention, to continue using it, and to use it as frequently or often as is needed to reap therapeutic benefits.

### Limitations

An important limitation of research on acceptability, as identified in the risk of bias assessment, lies with evidence generally being provided by patients who have agreed to use the digital mental health intervention and continued to engage with the study or the digital mental health intervention. Often missing from these findings is the acceptability of digital mental health interventions among individuals who had the opportunity to participate in a study involving a digital mental health intervention but chose not to. Nonparticipation and early termination in digital health have long prompted calls for more research attention on attrition, for example, Eysenbach [169] in 2005, along with related constructs such as adherence and engagement. There remains a need to establish consensus approaches to analyzing attrition, adherence, and engagement [170], and their relationship with acceptability. It is recommended that appropriate methods for such analyses be developed, to be used consistently in future evaluations of digital mental health interventions.

A second limitation comes from potential conflict in the research evidence between the acceptability of the intervention technology and the acceptability of the intervention as a whole. Future research should differentiate acceptability of the technology from the acceptability of other aspects, such as the timing or pacing of the intervention, or the treatment paradigm applied.

A further limitation is that the terminology used to identify acceptability is varied and changeable between studies. Data on acceptability not labelled as such may be difficult to integrate into acceptability research at large.

Finally, literature on this topic has increased exponentially following the COVID-19 pandemic [171]. Any review can only represent a snapshot of the available research at a point in time. At the same time, despite this proliferation of research, diverse populations (in terms of ethnicity, income and education levels, sexual and gender minority status, etc) remain underrepresented [172]. More evaluation studies are needed that ensure their inclusion and provide demographic data as appropriate, to further our understanding of their acceptability of digital mental health interventions.

### Conclusions

There is evidence for the acceptability of digital mental health interventions among patients with depression or anxiety disorders, with most studies in this review indicating positive acceptability. However, the available research evidence for acceptability is, as a whole, of only moderate quality. It is suggested that future intervention studies be planned from the outset to produce higher quality evidence for acceptability. Enhanced research evidence for acceptability will provide better insights into the appropriateness of digital mental health interventions for patients with depression or anxiety disorders.

## Appendix

Multimedia Appendix 1 Search strategies.

Multimedia Appendix 2 Acceptability assessment tool.

Multimedia Appendix 3 Description of acceptability results in the included studies.

Multimedia Appendix 4 PRISMA (Preferred Reporting Items for Systematic Reviews and Meta-Analyses) 2020 checklist.

Multimedia Appendix 5 PRISMA (Preferred Reporting Items for Systematic Reviews and Meta-Analyses) 2020 checklist for abstracts.

## Abbreviations

CBT: cognitive behavioral therapy
GRADE: Grading of Recommendations, Assessment, Development and Evaluation
MeSH: Medical Subject Headings
PRISMA: Preferred Reporting Items for Systematic Reviews and Meta-Analyses
PROSPERO: International Prospective Register of Systematic Reviews
RCT: randomized controlled trial
