# Supplementary material for: Acceptability of Digital Mental Health Interventions for Depression and Anxiety: Systematic Review
Source: J Med Internet Res. 2024 Oct 28;26:e52609. doi: 10.2196/52609 (PMC11555460; doi:10.2196/52609)
Supplement: Multimedia Appendix 1 [file jmir_v26i1e52609_app1.doc]

## Multimedia Appendix 1

## Search Strategies

## This is a Multimedia Appendix to a full manuscript published in the J Med Internet Res. For full copyright and citation information see http://dx.doi.org/10.2196/52609.

1. In PubMed:

("Depression"[MeSH Terms] OR "Depressive Disorder"[MeSH Terms] OR "depress*" OR "unipolar*" OR "dysthym*" OR "seasonal affective disorder" OR "postpartum depress*" OR "premenstrual dysphori*" OR "Anxiety"[MeSH Terms] OR "Anxiety Disorders"[MeSH Terms] OR "anxi* OR "agoraphobia"[MeSH Terms] OR "agoraphobia*" OR "panic"[MeSH Terms] OR "panic" OR "phobi*")

AND

("intervention"[Title/Abstract] OR "Psychosocial Intervention"[MeSH Terms])

AND

("wearable*" OR "smart*" OR "iphone*" OR "watch" OR "glass*" OR "personal digital assistant*" OR "handheld*" OR "tracker*" OR "microcomputers"[MeSH Terms] OR "microcomput*" OR "app" OR "apps" OR "mobile application*" OR "web application*" OR "Mobile Applications"[MeSH Terms] OR "Wearable Electronic Devices"[MeSH Terms] OR "monitoring, ambulatory"[MeSH Terms] OR "computers, handheld"[MeSH Terms] OR "Cell Phone"[MeSH Terms] OR "Video Games"[MeSH Terms] OR "Social Media"[MeSH Terms] OR "Internet-Based Intervention"[MeSH Terms])

AND

("acceptability" OR "acceptable" OR "satisfaction" OR "engagement" OR "self efficacy" OR "Patient Acceptance of Health Care"[MeSH Terms] OR "self efficacy"[MeSH Terms])

1. In Web of Science

(depression OR unipolar OR dysthym* OR "seasonal affective disorder" OR "postpartum depress*" OR "premenstrual dysphori*" OR anxiety OR agoraphobia OR panic OR phobi*)

AND

(wearable* OR "personal digital assistant" OR handheld OR tracker OR microcomputer OR "app" OR "apps" OR "mobile application" OR "web application"))

AND

("acceptability" OR "satisfaction" OR "engagement" OR "self-efficacy")

AND

(intervention (Title) or intervention (Abstract))

1. In Ovid

(depression OR unipolar OR dysthym* OR "seasonal affective disorder" OR "postpartum depress*" OR "premenstrual dysphori*" OR anxiety OR agoraphobia OR panic OR phobi* OR "Depression".mh. OR "Depressive Disorder".mh. OR "Anxiety".mh. OR "Anxiety Disorders".mh.)

AND

(wearable* OR "personal digital assistant" OR "mobile application" OR "web application" OR "Mobile Applications".mh. OR "Wearable Electronic Devices".mh. OR "Computers, Handheld".mh. OR "Video Games".mh. OR "Internet-Based Intervention".mh.)

AND

("acceptability" OR "satisfaction" OR "self-efficacy" OR "Patient Acceptance of Health Care".mh.)

AND

(intervention.ab. OR intervention.ti. OR "Psychosocial Intervention".mh.)
