# Supplementary material for: Acceptability of Digital Mental Health Interventions for Depression and Anxiety: Systematic Review
Source: J Med Internet Res. 2024 Oct 28;26:e52609. doi: 10.2196/52609 (PMC11555460; doi:10.2196/52609)
Supplement: Multimedia Appendix 2 [file jmir_v26i1e52609_app2.doc]

## Multimedia Appendix 2

## Acceptability Assessment Tool

## This is a Multimedia Appendix to a full manuscript published in the J Med Internet Res. For full copyright and citation information see http://dx.doi.org/10.2196/52609.

1. Definition:
   1. Is acceptability defined a priori? [1 point]
   2. If yes, does the definition reference prior research? [1 point]
2. Selection of quantitative measure(s):
   1. For the one or more acceptability measures mentioned, none are validated [1 point]
   2. For the one or more acceptability measures mentioned, all are validated [3 points]
   3. For the one or more acceptability measures mentioned, mix of validated and not validated [2 points]
3. Selection of qualitative instrument(s), if any:
   1. Were participants asked to provide open-ended feedback (i.e., in an interview or in a questionnaire seeking open-ended responses) on the technology? [2 points]
   2. If yes, are there one or more questions/prompts that target acceptability? [1 point]
4. Timing: Was acceptability measured at more than one time point (e.g., pre-test, during study period, post-test, follow-up)? [1 point]
5. Quantitative or qualitative instruments: are details provided in the paper or appendices for all questions/prompts given to participants? [2 points]
6. Quantitative results:
   1. Are descriptive statistics provided for all acceptability measures? [3 points; if some are provided but not all, then 1-2 points]
   2. If descriptive statistics are not provided for any acceptability measures, is a p-value provided with or without confidence intervals for any of the acceptability measures? [2 points]
7. Qualitative results: are descriptive summaries provided for responses to interview or open-ended survey questions? [1 point]

Points are summed to arrive at a total score out of 15.

Interpretation of total score: 0-15 (0-5 low, 6-11 moderate, 11-15 high)
