# Supplementary material for: Acceptability of Digital Mental Health Interventions for Depression and Anxiety: Systematic Review
Source: J Med Internet Res. 2024 Oct 28;26:e52609. doi: 10.2196/52609 (PMC11555460; doi:10.2196/52609)
Supplement: Multimedia Appendix 3 [file jmir_v26i1e52609_app3.docx]

**Multimedia Appendix 3**

**Table S1.** Description of acceptability results in the 41 RCTs on digital mental health interventions for depression.

| Author (Year) | Diagnosis | Intervention technology, program, treatment paradigm | Sample | Acceptability outcome | Acceptability results | Summarized acceptability result | LOE | Acceptability score |
| --- | --- | --- | --- | --- | --- | --- | --- | --- |
| Berger, Hämmerli, et al. (2011) [24] | MDD^a^ or dysthymia | Web-based program, Deprexis, CBT^b^ | N=76, unguided self-help 25, guided self-help 25, waitlist 26. Female: 53. | Global patient satisfaction (CSQ-8^c^ / ZUF-8^d^) | Mean CSQ-8 3.12 (SD 0.44) for guided self-help condition vs. unguided 2.86 (SD 0.53), *d*=0.54, ns, *F*_1,45_=3.26, *P*=.078. | Positive | 2 | 8 |
| Birney et al. (2016) [25] | Mild-to-moderate depression | Mobile app, MoodHacker, CBT | N=300, 150 MoodHacker, 150 depression info websites. Female: 230. Other than white: 85. | User satisfaction scale; program usability via SUS^e^_._ | Mean program satisfaction 4.6 (SD 1.0). Mean SUS at 6-week follow-up 79.7 (SD 17.1) | Positive | 2 | 6 |
| Blackwell et al. (2015) [26] | MDD | Web-based program, CBM^f^ | N=150, 76 imagery condition, 74 control. Female: 103. | Three Expectancy questions adapted from CEQ^g^. Satisfaction via three questions (1 to 7) | Six-month follow-up mean satisfaction imagery 4.79 (SD 1.32), control 3.89 (SD 1.57). Mean confidence recommending, imagery 5.24 (SD 1.31) vs. control 3.84 (SD 1.79). | Positive | 2 | 8 |
| Bücker et al. (2019) [27] | Mild-to-moderate depressive symptoms | Web-based program, MOOD, CBT | N=125, 62 MOOD, 63 Care-as-usual. Female: 92. | Expectation for treatment outcome (1 to 9); subjective appraisal adapted from ZUF-8; open questions for feedback. | Mean treatment expectation, MOOD 5.26 (SD 1.55), 5.63 (SD 1.42) care-as-usual. Overall positive subjective appraisals. | Positive | 2 | 8 |
| Buntrock et al. (2015) [28] | Subthreshold depression | Web-based program, GET.ON Mood Enhancer, CBT | N=406, 202 intervention, 204 web psychoeducation. Female: 300. Other than white: 65. | CEQ; user satisfaction per ZUF-8. | Mean satisfaction 26.27 (SD 5.32), 88% satisfied overall. | Positive | 2 | 9 |
| Burton et al. (2016) [29] | MDD | Computer-based program, Help4Mood, CBT | N=28, 14 Help4Mood, 14 TAU^h^. Female: 18. | Semi-structured interview | Almost all would use final program and would recommend, but inappropriate or inconvenient to use daily. | Mixed | 2 | 4 |
| de Graaf et al. (2009) [30] | Depression | Web-based program, Colour Your Life, CBT | N=303, 100 online CBT without support, 103 TAU, 100 online CBT without support, TAU by GP^i^. Female: 172. | CEQ plus separate questions on expectancy; satisfaction; evaluation questionnaire | Program only, 66% satisfied, 29% neutral, 5% dissatisfied; program with TAU, 71% satisfied, 25% neutral, 4% dissatisfied. | Mixed | 2 | 6 |
| Geraedts et al. (2014) [31] | Elevated depressive symptoms | Web-based program, Happy@Work, CT^j^ and PST^k^ | N=231, 116 Happy@Work, 115 care-as-usual. Female: 144. | Internet Intervention Evaluation Questionnaire (items 1-10 and open-ended questions). | Post-treatment mean satisfaction for website 7.4 (SD 0.9), intervention 7.4 (SD 1.2). | Positive | 2 | 8 |
| Gómez Penedo et al. (2020) [32] | Moderate depressive symptoms | Web-based program, Deprexis, CBT | N=223, all Deprexis and email support. Female: 157. | Patient satisfaction via adapted ZUF-8. | Mean ZUF-8 3.13 (SD 0.59). | Positive | 2 | 8 |
| Høifødt et al. (2013) [33] | Depression | Web-based program, MoodGYM, CBT | N=106, 52 MoodGYM, 54 waitlist. Female: 77. | Satisfaction with treatment via 9 questions. | 89% overall 4 or 5 of 5. 94% 4 or 5 on recommending. 50-60%, 4 or 5 on benefit and content usefulness and relevance. | Positive | 2 | 6 |
| Johansson et al. (2019) [34] | Depression | Web-based program, CBT | N=54, 27 therapist-supported iCBT^l^, 27 waitlist. |  | Acceptance rate (satisfied or very satisfied) 89%. | Positive | 2 | 4 |
| Kelders et al. (2015) [35] | Mild to moderate depressive symptoms | Web-based program, Living to the Full, ACT^m^ | N=239, 113 Automated Support, 126 Human Support. Female: 169. Ethnicity other than Dutch, 21. | Task enjoyment (5 items), involvement (shortened Personal Involvement Inventory, 10 items), and trust. | Mean task enjoyment 5.83 (SD 1.16), involvement 5.69 (SD 1.14), satisfaction 4.21 (SD 0.81). | Positive | 2 | 9 |
| Kenter et al. (2016) [36] | MDD | Web-based program, PST | N=269, 136 intervention, 133 self-help book with no guidance (control). Female: 145. | General satisfaction: CSQ-8. Alles Onder Controle satisfaction questionnaire. | Overall mean 7 of 10 (SD 1.08). 75% useful, 32% very or mostly, 8% not helpful. Mean CSQ-8 20.4 (SD 6.1). | Positive | 2 | 6 |
| G. Kok et al. (2014) [37] | Remission for recurrent MDD | Web-based program, Mobile CT, CT | N=129, all Mobile CT + TAU condition. Female: 102. | After each module, perceived usefulness and perceived difficulty, plus qualitative experiences. | Most rated modules 1-7 useful, 8 very useful. Most rated all modules as easy. Some described as helpful and easy to use. | Positive | 2 | 5 |
| Lappalainen et al. (2015) [38] | Depressive symptoms | Web-based program, Good Life Compass, ACT | N=39, 19 internet-based ACT, 20 waitlist. Female: 28. | Visual Analogue Scale (1-10); treatment satisfaction (1-10); recommending to others with similar symptoms (1-10); interview questions. | Mean satisfaction 8.22 (SD 1.59). Overall positive comments on satisfaction. Mean recommending 8.94 (SD 0.10) with positive open-ended responses. | Positive | 2 | 11 |
| Levin et al. (2011) [39] | Depression | Computer-based program (CD-ROM), Wellness Workshop, CBT | N=191, 100 treatment (TAU plus Wellness Workshop), 91 control (TAU). Female: 148. Other than white: 19. | Post-assessment survey (program contents and effects). | Program friendly and easy to use (mean 4.9, SD 1.3), relevant to needs (4.4, SD 1.4), helped achieve changes (4.0, SD 1.3), would recommend (4.6, SD 1.5), useful (4.5, SD 1.4). Overall 3.8 of 5 (SD 0.9). | Positive | 2 | 4 |
| Löbner et al. (2018) [40] | Mild to moderate depression | Web-based program, MoodGYM, CBT | N=647, 327 control group (TAU), 320 intervention group (MoodGYM + TAU). Female: 443. | Shortened USE (items 1-7). | Intervention mean USE 4.7 (95% CI 4.5 to 4.8) of 7 at 6 weeks, 4.6 (95% CI 4.4 to 4.8) at 6 months. | Positive | 2 | 9 |
| Lüdtke et al. (2018) [41] | Depressive symptoms | Mobile app, Be Good to Yourself, CBT | N=88, 44 intervention group, 44 waitlist. Female: 69. | CSQ-8 (adapted ZUF-8) for those who used app | ZUF-8 items positive at 57.7%-88.5%. | Positive | 2 | 8 |
| Lukas & Berking (2021) [42] | Depressive symptoms | Mobile app, mentalis Phoenix (MT Phoenix), CBM | N=16, 5 MT Phoenix app, 11 waitlist. Female: 13. | Acceptance and usability via SUS at post-assessment. | Mean SUS 91.25 (SD 2.06). | Positive | 2 | 8 |
| Lukas et al. (2021) [43] | Depressive symptoms | Mobile app, mentalis Phoenix (MT Phoenix), CBM | N=77, 40 MT Phoenix, 37 waitlist. Female: 56. | Post-assessment perceived helpfulness (0-4). | Psychoeducation mean 3.17 (SD 0.72), approach-avoidance modification 3.00 (SD 0.53), behavioral activation tasks 3.17 (SD 0.55). | Positive | 2 | 6 |
| Ly et al. (2014) [44] | MDD | Mobile app, behavioural activation and mindfulness | N=81, 40 behavioural activation, 41 mindfulness. Female: 57. | Credibility/expectancy scale at end of week one. | Mean credibility /expectancy score for behavioural activation 31.9 of 50 (SD 7.1), mindfulness 32.1 (SD 7.8). | Positive | 2 | 8 |
| Ly, Topooco, et al. (2015) [45] | MDD | Mobile app, behavioural activation | N=93, 46 blended treatment (in-person & app), 47 full behavioural activation (in-person & homework). Female: 65. | Credibility/expectancy scale, 5 items on 10-point scale, at end of first week of treatment. | Mean credibility /expectancy score for blended 33.0 of 50 (SD 8.0), full behavioural activation 33.5 (SD 6.5). | Positive | 2 | 8 |
| Meyer et al. (2009) [46] | Depression | Web-based program, Deprexis, CBT | N=396, 320 Deprexis plus care as usual, 76 control (waitlist). Female: 301. | Questions on overall impression, subjective benefit, user satisfaction, content quality, recommending to others. | 83% overall rating 1-3 of 6, 82% helps at least a bit, 78% met or exceeded expectations, 95% would recommend for mild depression (79% moderate, 42% severe). | Positive | 2 | 6 |
| Meyer et al. (2015) [47] | Severe depression | Web-based program, Deprexis, CBT | N=163, 78 Deprexis plus care as usual, 85 control (care as usual/waitlist). | Satisfaction at post-treatment via adapted ZUF-8; alliance/helpfulness via adapted Helping Alliance Questionnaire three weeks after randomizing. | Mean total ZUF-8 24.17 (SD 4.82). 83.3% would recommend, 88.3% program quality good or excellent, 71% positive helpfulness. | Positive | 2 | 9 |
| Moritz et al. (2012) [48] | Depression | Web-based program, Deprexis, CBT | N=210, 105 Deprexis, 105 waitlist. Female: 165. | Questions on feasibility, effectiveness, perceived quality, applicability; adapted ZUF-8. | Comprehensibility and other aspects rated good. Every second participant reported reduced symptoms. Most recommend for mild depression, minority would for severe depression. | Positive | 2 | 8 |
| Motter et al. (2019) [49] | Depressive symptoms | Mobile app, cognitive training | N=46, 21 verbal ability, 25 executive function/processing speed. Female: 33. Black, Asian, Hispanic, Other: 25. | At week 8, User Engagement Scale (1-7). | Groups did not significantly differ on any subscales. Those with low training load (<150 min) did not significantly differ from those with high training load (≥150 min). | Not enough information | 2 | 8 |
| Oehler et al. (2020) [50] | Depression or dysthymia | Web-based program, iFightDepression, CBT | N=347, 173 intervention group, 174 control (progressive muscle relaxation). Female: 273. | ZUF-8 adapted for web-based interventions. | Mean total CSQ-8 25.31 of 32 for intervention vs. control 21.97. | Positive | 2 | 8 |
| Perini et al. (2009) [51] | Depression | Computer-based program, Sadness Program, CBT | N=45, 27 treatment group, 18 waitlist. Female: 35. |  | 82% very or mostly satisfied, neutral or somewhat 18%. 94% excellent or good module quality. Mean confidence in treatment 7 of 10, confidence recommending 8. | Positive | 2 | 4 |
| Pinto et al. (2015) [52] | Depression | Computer-based program, eSMART-MH | N=28, 12 eSMART-MH, 16 attention control with education modules. Female: 67%. African American: 67%. | Open-ended questions about the program. | Overall, content was liked. Ideas for revisions provided. | Positive | 2 | 6 |
| Reins et al. (2019) [53] | MDD | Web-based program, GET.ON Mood Enhancer (shortened), CBT | N=131, 65 iCBT, 66 online psychoeducation. Female: 99. | User satisfaction via CSQ-8. | For iCBT, 83% overall satisfaction, 92.6% high quality, 75.9% had needs met, 88.9% would use program again as needed, 87.0% would recommend. | Positive | 2 | 7 |
| Ruwaard et al. (2009) [54] | Mild to moderate depression | Web-based program, CBT | N=54, 36 web-based CBT, 18 waitlist. Female: 37. | Posttreatment evaluation of overall value, scale of 1 to 10. | Mean overall value of treatment 7.7 (SD 1.2). | Positive | 2 | 4 |
| Smith et al. (2017) [55] | MDD | Web-based program, Sadness Program, CBT | N=270, 61 iCBT, 141 different self-help books, 68 waitlist. Female: 203. | Pretreatment expectancy: logic and expected success; posttreatment satisfaction and confidence recommending to friend. | Pretreatment, iCBT mean logic 5.81 (SD 2.10), expected success 5.28 (SD 1.65). Posttreatment mean satisfaction 6.45 (SD 1.84), confidence recommending 6.73 (SD 2.32). | Positive | 2 | 7 |
| Stephens (2014) [56] | Depressive symptoms | Web-based program, CBM | N=124, 66 training group (CBT emotion recognition task), 58 control (sham emotion recognition task). Female: 103. | Acceptability measure [57] at post-intervention. | Training group, 60% would use again and 40% would recommend to friend; 68% found it too long; 52-64% found task not enjoyable or engaging, or enjoyment or engagement decreased. | Mixed | 2 | 6 |
| Titov, Andrews, Davies, et al. (2010) [58] | Depression | Web-based program, Sadness Program, CBT | N=127, 41 technician-assisted group, 46 clinician-assisted group, 45 control group (waitlist/delayed treatment). Female: 94. | At pre-treatment, CEQ expectancy factors. At post-treatment, treatment satisfaction based on CEQ. | 87% very or mostly satisfied, 13% neutral or somewhat dissatisfied. Mean confidence recommending 8 of 10. | Positive | 2 | 9 |
| Titov et al. (2015) [59] | MDD (and comorbid anxiety disorders) | Web-based program, Wellbeing Course and Mood Course, CBT | N=290, 149 transdiagnostic CBT, 141 disorder-specific CBT. Female: 208. | Two post-treatment questions: confidence recommending treatment and whether it was worth the time (Yes/No responses) | 92% of transdiagnostic and 96% of disorder-specific CBT groups would recommend, 96% and 97% of these groups found it worth their time. | Positive | 2 | 6 |
| Tønning et al. (2021) [60] | Depression | Mobile app, Monsenso, Monitoring and CBT | N=120, 59 Monsenso, 61 standard treatment by treating physician. Female: 63. | Satisfaction via Verona Satisfaction Scale-Affective Disorder. | Intervention group more satisfied vs. control, once adjusted for baseline values, psychiatric center, admittances, age, sex, and depression score, B=7.80, 95% CI: -0.025; 15.63, *P*=0.051. | Not enough information | 2 | 7 |
| Wahbeh (2018) [61] | Depression symptoms | Web-based program, Internet Mindfulness Meditation Intervention, mindfulness | N=50, 26 internet mindfulness meditation intervention, 24 waitlist control. Female: 40. Caucasian: 42. | CSQ-8 at posttreatment | Mean 2.54 ± 0.21 on 1-4 scale. Open text had 16 positive responses, one positive and negative, three neutral, and four negative. | Positive | 2 | 11 |
| Watts et al. (2013) [62] | MDD | Mobile app and web-based program, Sadness Program and Get Happy Program, CBT | N=35, 15 Mobile group, 20 Computer group. Female: 28. | At posttreatment, 2 items based on CEQ. | 54% of Mobile Group and 64% of Computer Group were very satisfied, remaining somewhat satisfied. 64% of both groups very confident recommending, remaining somewhat confident. | Positive | 2 | 7 |
| Williams et al. (2013) [63] | Major depressive episode | Web-based program, Sadness Program, CBT and CBM | N=63, 35 cognitive bias modification plus iCBT, 28 waitlist. Female: 48. | Questionnaire adapted from CEQ. | Mean baseline treatment expectancy 3.22 (SD 0.80). Mean confidence recommending 7.77 (SD 2.10). | Positive | 2 | 7 |
| Williams et al. (2015) [64] | MDD | Web-based program, Sadness Program, CBT and CBM | N=75, 36 positive condition (CBM plus iCBT), 39 control. Female: 55. Australia/New Zealand born: 60. | Treatment Expectancy (adapted from CEQ) and Outcomes Questionnaire at baseline and posttreatment. | For positive condition, expectancy 3.30 (SD 0.89); 25% dissatisfied or neutral and 75% mostly or very satisfied. Mean confidence recommending 7.18 of 10 (SD 1.95). | Positive | 2 | 7 |
| Wong et al. (2021) [65] | Depressive symptoms | Mobile app, Lifestyle Hub, transtheoretical model | N=79, 39 lifestyle medicine group, 40 waitlist control group. Female: 67. | CEQ | No significant difference in credibility, t_38_=–1.72, *P*=.09, and expectancy, t_38_=–0.90, *P*=.37, from baseline to Week 9, and from Week 9 to 13 (*P*>.05). | Not enough information | 2 | 7 |

^a^MDD: major depressive disorder

^b^CBT: cognitive behavioural therapy

^c^CSQ: Client Satisfaction Questionnaire

^d^ZUF: Fragebogen zur Messung der Patientenzufriedenheit, German version of CSQ

^e^SUS: System Usability Scale

^f^CBM: cognitive bias modification

^g^CEQ: Credibility/Expectancy Questionnaire

^h^TAU: treatment as usual

^i^GP: general practitioner

^j^CT: cognitive therapy

^k^PST: problem-solving therapy

^l^iCBT: internet-based cognitive behavioural therapy

^m^ACT: acceptance and commitment therapy

**Table S2.** Description of acceptability results in the 23 non-RCT quantitative studies on digital mental health interventions for depression.

| Author (Year) | Diagnosis | Intervention technology, program, treatment paradigm | Sample | Acceptability outcome | Acceptability results | Summarized acceptability result | LOE | Acceptability score |
| --- | --- | --- | --- | --- | --- | --- | --- | --- |
| Addington et al. (2019) [66] | Elevated depressive symptoms | Web-based program, MARIGOLD, positive psychology | Phase 1: N=58, intervention 26, emotion reporting 17, waitlist 15. Female: 38. Other than white: 24. | Interview questions on content; survey on recommending the program; open-ended questions. | Phase 1: 100% expectations met or exceeded, helpful or extremely helpful; none dissatisfied overall. Phase 2: 95% expectations met, one (2.5%) dissatisfied overall. | Positive | 3 | 7 |
| Berman et al. (2014) [67] | Depression | Computer-based program, ePST, PST^a^ | N=26, all intervention group. Female: 79%. Native American: 1. | SUS^b^, acceptability of self-guided treatment, credibility questionnaire. | Mean SUS 79.4 (SD 17.3). Mean week 4 credibility 6.7 (SD 1.9), week 10 7.5 (SD 2). Mean week 4 acceptability 5.29 (SD 1.1), week 10 5.46. | Positive | 3 | 8 |
| Burns et al. (2011) [68] | MDD^c^ | Mobile app, Mobilyze!, ecological momentary assessment | N=8, 8 treatment. Female: 7. Non-Hispanic Caucasians: 7. | Satisfaction rated 1 to 7; semistructured interview (created ad-hoc). | Mean satisfaction 5.71 (SD 1.38). | Positive | 3 | 8 |
| Callan et al. (2021) [69] | MDD with at least mild-to-moderate depressive symptoms | Mobile app, CBT MobileWork, CBT^d^ | Phase 1: N=8, all treatment group. Female: 7. Caucasian: 7. Phase 2: N=15, all treatment group. Female: 11. Other than white: 3. | Phase 1: After-Scenario Questionnaire. Post-Study Satisfaction and Usability Questionnaire. Phase 2: Computer System Usability Questionnaire, adapted TAM^e^. Post-study interview. | Phase 1: Mean After-Scenario Questionnaire 1.63 (SD 1.00) session one and 1.76 (SD 1.49) session two. Mean Post-Study Satisfaction and Usability Questionnaire 1.36 (SD 0.31). Phase 2: mean Computer System Usability Questionnaire 66.00 (SD 26.0). Mean baseline TAM 2.02 (SD 0.62). | Positive | 3 | 12 |
| Caplan et al. (2018) [70] | Mild-to-moderate depressive disorder | Mobile app, VoiceThread, CBT | N=18, all treatment group. Female: 15. | Open-ended questionnaire via interview; Likert-type questions on acceptability of contents. | Almost all interested in mobile app therapy. Most found content relevant, comprehensible, and culturally appropriate. | Positive | 3 | 7 |
| Cartreine et al. (2012) [71] | Depression | Computer-based program, ePST, PST | N=14, 7 ePST, 7 waitlist. Female: 10. Black: 1, Hispanic: 2. | SUS, Credibility Questionnaire, Assessment of Self-Guided Treatment | ePST group mean SUS 80.36 of 100 (SD 19.28) after session 1, 85.36 (SD 15.91) after session 6. Mean Credibility Questionnaire items 7.14-8.43 (SD 1.27-2.82). Mean Assessment of Self-Guided Treatment items 4.14-6.43 (SD 0.38-1.72). | Positive | 3 | 9 |
| Coutinho et al. (2021) [72] | Depression | Web-based program, POLYHYMNIA Mood | N=24, 12 dynamic playlist, 12 static playlist. | Questions on ease of use, perceived effectiveness, recommending to friends and family. | Mean usability 'easy' 4.1 of 5, perceived effectiveness 7 of 10, recommending 9.1 of 10. | Positive | 3 | 6 |
| Dahne et al. (2018) [73] | Elevated depressive symptoms | Mobile app, Behavioral Apptivation, behavioural activation | N=11, all Behavioral Apptivation. Female: 10. Black, Asian, or Other: 6. | Posttreatment questions on experiences. | Mean ease of use 4.00 (SD 0), desire to keep using app 2.80 (SD 0.92), benefits from use 3.80 (SD 0.92). | Positive | 3 | 6 |
| Dear et al. (2013) [74] | Depression | Web-based program, Managing Your Mood, CBT | N=20, all treatment group. Female: 13. | Treatment satisfaction at posttreatment via two questions, yes/no responses. | 82% would recommend course and 82% found program worth their time. | Positive | 3 | 6 |
| Drake et al. (2013) [75] | Mild-to-moderate depression | Web-based program, Moodscope, Mood tracking | N=20, all Moodscope. Female: 15. Black: 2. | Moodscope Usability and Usefulness Questionnaire with structured and open-ended questions. | Mean helpfulness 5.6/10 (SD 2.28). ‘Too time-consuming’ 6.4 (SD 2.77). One found tracking too infrequent, 3 adequate, 3 slightly too frequent, 7 too frequent. | Mixed | 3 | 8 |
| Fuller-Tyszkiewicz et al. (2018) [76] | Depression | Mobile app, BlueWatch, CBT | N=5, all BlueWatch. Female: 4. | Perceived usability per SUS and open-ended questions. | Mean SUS 86.00 (SD 10.84) | Positive | 3 | 12 |
| Gould et al. (2021) [77] | Elevated depressive symptoms | Mobile app, Meru Health Program | N=20, all Meru Health Program. Female: 14. Other than white: 8. | User experience survey, 6 items; interviews. | 79% expectations somewhat or fully met, 89% helpful, and 85% easy to use. | Positive | 3 | 8 |
| Harper Shehadeh et al. (2020) [78] | Depression | Web-based program, Step-by-Step | N=126, all Step-by-Step. Female: 100. | Post-assessment satisfaction questions based on CSQ^f^ | Mean total satisfaction 26.5 of 35. | Positive | 3 | 11 |
| Jacmon et al. (2010) [79] | Mild-to-moderate depression | Web-based program, CBT | N=9, all iCBT & in-person CBT as needed. Female: 4. | Treatment Evaluation Questions (created ad-hoc). | Intervention valuable per all six completers. | Positive | 4 | 5 |
| Kooistra et al. (2016) [80] | Depression | Web-based program, Minddistrict, CBT | N=9, all blended CBT. Female: 5. | Pre-treatment CEQ^g^. Posttreatment usability via SUS, satisfaction via CSQ-8. | Mean SUS 73.2 (SD 7.3). Mean CSQ-8 22.7 (SD 4.8). One somewhat and 6 mostly or very satisfied. | Positive | 3 | 12 |
| Mayer et al. (2022) [81] | Depression (and those without) | Mobile app, SELFPASS, Mood tracking | N=41 (participants with depression), all SELFPASS. Female: 30. | Acceptance after 5 days of use via open text fields on user experience. | Mean perceived program quality 1.17 (SD 0.66), practicality 0.92 (SD 0.67), acceptance 0.51 (SD 0.86). | Positive | 3 | 11 |
| Schlosser et al. (2017) [82] | Depression | Mobile app, PRIME-D | N=36, all PRIME-D. Female: 28. | Self-report satisfaction measure (created ad-hoc); qualitative feedback; survey (optional at 4 and 8 weeks). | Post-treatment mean satisfaction 7.23 (SD 2.8). At 4 weeks and 8 weeks, 83% would recommend app (no change). | Positive | 3 | 6 |
| Schuster et al. (2019) [83] | Depression | Web-based program and mobile app, Minddistrict, ACT^h^ | N=27, all blended group therapy. Female: 14. | SUS and ZUF-8^i^ | Mean SUS 65.33 of 100 (SD 18.95). Mean ZUF-8 26.43 of 32 (SD 4.80). | Positive | 3 | 8 |
| Stiles-Shields et al. (2019) [84] | Depression | Mobile app, Boost Me (behavioural activation)and Thought Challenger, (CT^j^) | N=30, 10 Boost Me, 10 Thought Challenger, 10 waitlist. | SUS at weeks 3 and 6 for Boost Me and Thought Challenger participants. | Week 3 mean SUS: Thought Challenger (84.10 ± 10.43) and Boost Me (70.00 ± 14.31). Week 6 post-treatment mean SUS: Thought Challenger (88.57 ± 5.56) and Boost Me (78.33 ± 15.10). | Positive | 3 | 9 |
| Strauss et al. (2021) [85] | Depression | Mobile app, Headspace, Mindfulness | N=54, all Headspace. Female: 37.Other than white:: 30. | Open-ended questions via Change Interview [86]. Baseline CEQ, SUS | Mean CEQ credibility subscale 6.26 of 9 (SD 1.40). | Positive | 3 | 11 |
| Tomasino et al. (2017) [87] | Depression | Web-based program, MoodTech, CBT | N=47, 12 individual internet intervention, 23 internet intervention with peer support, 12 waitlist. | SUS for overall usability and USE for usefulness, satisfaction, ease of use, ease of learning. | Mean SUS 68.75 (SD 16.09) internet intervention only, 60.5 (SD 16.69) with peer support. USE mean usefulness 4.39 (SD 1.45) for internet intervention and 4.05 (SD 1.3) with peer support, mean satisfaction 4.48 (SD 1.8) and 3.52 (SD 1.31) respectively. | Positive | 3 | 8 |
| Walsh (2018) [88] | Depression | Web-based program, Uplift, positive psychology | N=103, all Uplift. Female: 74. First language is English: 84. | At follow-up, acceptability via three items, on 7-point Likert scale; open-ended comments | 43% enjoyable, and 37% difficult. 20% rated as helpful. 39% satisfied overall, 32% were in the middle, and 29% were dissatisfied. | Mixed | 3 | 9 |
| Yeung et al. (2018) [89] | Depression | Web-based program, MoodGYM, CBT | N=75, 37 intervention group (MoodGYM & TAU^k^), 38 TAU. Female: 77%. | Satisfaction via 8-item ad-hoc scale | 65% easy to use, 64% too long, 63% useful in improving mood, 36% would recommend. | Mixed | 2 | 6 |

^a^PST: problem-solving therapy

^b^SUS: System Usability Scale

^c^MDD: major depressive disorder

^d^CBT: cognitive behavioural therapy

^e^TAM: Technology Acceptance Model

^f^CSQ: Client Satisfaction Questionnaire

^g^CEQ: Credibility/Expectancy Questionnaire

^h^ACT: acceptance and commitment therapy

^i^ZUF: Fragebogen zur Messung der Patientenzufriedenheit, German version of CSQ

^j^CT: cognitive therapy

^k^TAU: treatment as usual

**Table S3.** Description of acceptability results in the 3 qualitative studies on digital mental health interventions for depression.

| Author (Year) | Diagnosis | Intervention technology, program, treatment paradigm | Sample | Acceptability outcome | Acceptability results | Summarized acceptability result | LOE | Acceptability score |
| --- | --- | --- | --- | --- | --- | --- | --- | --- |
| Knowles et al. (2015) [91] | Depression | Web-based program, MoodGYM and Beating the Blues, CBT^a^ | N=36. Female: 26. Other than white: 0. | Semi-structured interview | Seventeen participants ambivalent, 10 negative, and nine positive. | Mixed | 2 | 5 |
| Ly, Janni, et al. (2015) [92] | MDD^b^ | Mobile app, behavioural activation and mindfulness | N=12, all participants of [46]. Female: 6. | Semi-structured interviews. | Overall experience positive (n=5), neutral (n=4), and negative (n=3). | Mixed | 2 | 6 |
| J. Schneider et al. (2014) [90] | Depression | Web-based program, MoodGYM, CBT | N=637, 318 MoodGYM, 319 control (websites with mental health information). Female: 328. | Questionnaire including open-ended responses. | Negative and positive comments in 2.5 to 1 ratio. Reasons for negative experience varied, related and unrelated to treatment characteristics. | Mixed | 2 | 10 |

^a^CBT: cognitive behavioural therapy

^b^MDD: major depressive disorder

**Table S4.** Description of acceptability results in the 43 RCTs on digital mental health interventions for anxiety.

| Author (Year) | Diagnosis | Intervention technology, program, treatment paradigm | Sample | Acceptability outcome | Acceptability results | Summarized acceptability result | LOE | Acceptability score |
| --- | --- | --- | --- | --- | --- | --- | --- | --- |
| Allen et al. (2016) [93] | PD^a^ | Web-based program, Panic Program, CBT^b^ | Study 1 only. N=67, intervention 30, waitlist 37. | Satisfaction with online course (1-5); confidence recommending to friend (1- 10). | Mean satisfaction 4.53 (SD 0.83), 93% very/mostly satisfied. Mean confidence recommending 9.20 (SD 1.15), 87% mostly or very confident. | Positive | 2 | 6 |
| Bell et al. (2012) [94] | SAD^c^, PD, GAD^d^ | Web-based program, CRUfAD, CBT | N=83, intervention 40, waitlist 43. Female: 56. | Questions on program satisfaction (0-8) and treatment acceptability (0-8) | Mean satisfaction 5.6 (SD 1.5), mean acceptability 5.9 (SD 1.8). | Positive | 2 | 5 |
| Berger et al. (2009) [95] | SAD | Web-based program, CBT | N=52, intervention 31, waitlist 21. Female: 29. | Questions on satisfaction | 63% completely satisfied, 22% moderately satisfied, 11% not satisfied. | Positive | 2 | 5 |
| Berger, Caspar, et al. (2011) [96] | SAD | Web-based program, CBT | N=81, pure self-help 27, guided self-help 27, step-up of support on demand 27. Female: 43. | Global patient satisfaction (CSQ-8^e^ / ZUF-8^f^) | Mean CSQ-8 3.27 (SD .53), between somewhat and very satisfied. | Positive | 2 | 8 |
| Berger et al. (2014) [97] | SAD, PD with or without agoraphobia, and GAD | Web-based program, CBT | N=132, tailored treatment 44, disorder-specific treatment 44, waitlist 44. Female: 74. | Global patient satisfaction (CSQ-8) | Mean CSQ-8 3.34 (SD 0.45) for tailored condition. Disorder-specific group, mean CSQ-8 3.17 (SD 0.49). | Positive | 2 | 8 |
| Boettcher et al. (2013) [98] | SAD | Web-based program, CBM^g^ | N=129, 86 across four attention bias modification conditions, 43 across two control conditions. Female: 82. | Question on satisfaction (1-4) | Mean satisfaction 2.70 of 4 (SD .72). | Positive | 2 | 6 |
| Boettcher, Åström, et al. (2014) [99] | SAD, GAD, PD, or anxiety disorder not otherwise specified | Web-based program, mindfulness | N=91, 45 mindfulness-based treatment, 46 online discussion forum. Female: 65. | Question on satisfaction with treatment (1 to 5). | Mean satisfaction for mindfulness group 3.7 (SD 1.0). | Positive | 2 | 6 |
| Boettcher, Hasselrot, et al. (2014) [100] | SAD | Web-based program, CBT and CBM | N=133, 66 iCBT^h^ + attention training, 67 iCBT + control training. Female: 85. | Satisfaction with combined treatment ( rated 1 to 4). | Mean satisfaction 3.13 (SD 0.70). | Positive | 2 | 6 |
| Carlbring et al. (2001) [101] | PD | Web-based program, CBT | N=41. Female: 29. | TCS^i^; questionnaire on participant responses to the program. | Mean TCS score 42.6 of 50 (SD 5.6). | Positive | 2 | 10 |
| Carlbring et al. (2003) [102] | PD | Web-based program, CBT | N=22, 11 CBT, 11 applied relaxation. Female: 15. | TCS; questionnaire on participant responses to the program. | Mean TCS 34.9 for CBT (SD 9.5) and 32.6 for applied relaxation (SD 7.4) | Positive | 2 | 10 |
| Carlbring et al. (2005) [103] | PD | Web-based program, CBT | N=49, 24 Live therapy, 25 Internet-based therapy. Female: 35. Latin American: 1. | TCS; questionnaire on participant responses to the program. | Mean TCS 33.4 (SD 7.3) internet therapy conditions. Most satisfied or very satisfied. | Positive | 2 | 10 |
| Carlbring, Bohman, et al. (2006) [104] | PD | Web-based program, CBT | N=60, 30 Internet-based bibliotherapy, 30 waitlist. Female: 36. | TCS, questionnaire evaluating the program (including satisfaction) | Mean TCS 36.9 (SD 11.5). 21% satisfied, 76% very satisfied, 3% indifferent or dissatisfied. | Positive | 2 | 9 |
| Carter et al. (2013) [105] | GAD, PD, or SAD | Computer-based program, CLIMATE, CBT | N=83, 40 computerized CBT, 43 waitlist. | Questions on treatment credibility at baseline (1-7), treatment satisfaction (0-8), acceptability of computerised approach (0-8). | Mean treatment credibility 4.8-6.5 (SD 0.8-1.5). Mean treatment satisfaction 5.4-6.5 (SD 1.4-1.6). Mean treatment acceptability 5.3-6.0 (SD 1.5-2.3). | Positive | 2 | 7 |
| Dear et al. (2015) [106] | GAD (and comorbid disorders) | Web-based program, Worry Course and Wellbeing Course, CBT | N=338. 170 transdiagnostic CBT (Wellbeing Course), 168 disorder-specific CBT (Worry Course). 168 clinician-guided, 170 self-guided. Female: 256. | Treatment satisfaction and acceptability at post-treatment via two questions with a yes/no answer. | 99% of transdiagnostic and 97% of disorder-specific groups would recommend; 97% and 98% of these groups found course worth their time. | Positive | 2 | 6 |
| Dear et al. (2016) [107] | SAD (and comorbid disorders) | Web-based program, Social Confidence Course and Wellbeing Course, CBT | N=220. 105 transdiagnostic CBT (Wellbeing Course), 115 disorder-specific CBT (Social Confidence Course). 112 clinician-guided, 108 self-guided. Female: 128. | Treatment satisfaction and acceptability at post-treatment via two questions with a yes/no answer. | 94% of transdiagnostic and 95% of disorder-specific CBT groups would recommend; 94% and 97% of these groups found course worth their time. | Positive | 2 | 6 |
| Ebenfeld et al. (2021) [108] | PD with or without agoraphobia | Mobile app, GET.ON Panic, CBT | N=92, 45 GET.ON Panic, 47 waitlist. Female: 51. Asian or NA: 16. | User satisfaction from adapted ZUF-8. Usability via SUS^j^ at postassessment. | Mean SUS 71.16 (SD 18.97) of 100. Mean user satisfaction 28.10 of 32 (SD 5.09). | Positive | 2 | 8 |
| Fogliati et al. (2016) [109] | PD (and comorbid disorders) | Web-based program, Wellbeing Course and Panic Course, CBT | N=145, 72 transdiagnostic CBT, 73 disorder-specific CBT. 72 clinician-guided CBT, 73 self-guided CBT. Female: 115. | Treatment satisfaction and acceptability at post-treatment via two questions with a yes/no answer. | 98% of transdiagnostic and 98% of disorder-specific CBT groups would recommend. 93% and 95% of these groups found it worth their time. | Positive | 2 | 6 |
| Furmark et al. (2009) [110] | SAD | Web-based program, CBT | Trial 1 N=120, 40 iCBT, 40 bibliotherapy only, 40 waitlist. Female: 81. Trial 2 N=115, 29 iCBT, 57 bibliotherapy only or with discussion group, 29 internet applied relaxation. Female: 78. | After week one, 5 questions on perceptions of credibility (total 5-50). | Mean credibility 34.84 (SD 7.84) for iCBT, bibliotherapy alone 33.74 (SD 7.69), bibliotherapy & discussion 35.30 (SD 7.82), internet applied relaxation 36.35 (SD 7.45). | Positive | 2 | 5 |
| Gallego et al. (2011) [111] | SAD (fear of public speaking) | Web-based program, Talk to Me, CBT | N=41, 24 Talk to Me and 17 waitlist. Female: 28. | Motivation and satisfaction (each 0-10). | Baseline motivation 8.96 (SD 0.91). Completers were generally satisfied. | Positive | 2 | 7 |
| Hedman et al. (2011) [112] | SAD | Web-based program, CBT | N=126, 64 iCBT and 62 cognitive behavioural group therapy. Female: 45. | TCS | Mean credibility 34.0 (SD 9.03) for iCBT. | Positive | 2 | 8 |
| Johnston et al. (2011) [113] | GAD, SAD, or PD with or without agoraphobia | Web-based program, Anxiety Program, CBT | N=139, 43 coaching-assisted iCBT, 46 clinician-assisted iCBT, 42 waitlist. Female: 77. | Questions adapted from CEQ^k^ at post-treatment, satisfaction questions at follow-up. | 84% very or mostly satisfied, 16% neutral/somewhat dissatisfied, none very dissatisfied. 97% confident recommending. | Positive | 2 | 8 |
| Johnston (2012) [114] | Primary diagnosis of GAD, SAD, PD/agoraphobia | Web-based program, Anxiety Program, CBT | N=78, 40 treatment, 38 waitlist. Female: 53. | Questions on treatment satisfaction at post-treatment | 71% very or mostly satisfied, 29% neutral/somewhat satisfied, none dissatisfied. | Positive | 2 | 4 |
| Jones et al. (2016) [115] | GAD (clinical or subclinical) | Web-based program, GAD Online for Older Adults, CBT | N=46, 24 iCBT, 22 waitlist. Female: 40. | CEQ | Mean credibility 20.86/27 (SD 3.99), expectancy 18.13/29 (SD 4.34). | Positive | 2 | 6 |
| Kiropoulos et al. (2008) [116] | PD with or without agoraphobia | Web-based program, Panic Online, CBT | N=86, 46 Panic Online, 40 face-to-face CBT. Female: 62. | TCS-M at baseline; TSQ^l^; three other items. | Panic Online mean TCS 38.56 (SD 6.91), TSQ 5.76 of 7 (SD 2.99). | Positive | 2 | 8 |
| Klein et al. (2006) [117] | PD with or without agoraphobia | Web-based program, Panic Online, CBT | N=55, 19 Panic Online, 18 manualized CBT workbook, 18 information control-waitlist. Female: 44. | TCS-M at baseline; TSQ; written responses relating to program ratings. | Panic Online mean TCS-M 38.5 (SD 8.2). | Positive | 2 | 8 |
| Klein et al. (2009) [118] | PD with or without agoraphobia | Web-based program, Panic Online, CBT | N=57, 28 Panic Online and frequent contact, 29 Panic Online and infrequent contact. Female: 47. | Baseline TCS (0-50); TSQ. | Mean TCS 39.21 (SD 8.45) for frequent contact and 40.00 (SD 6.12) infrequent contact. | Positive | 2 | 8 |
| R. Kok et al. (2014) [119] | SAD, agoraphobia with or without PD, and/or specific phobia | Web-based program, Phobias Under Control | N=212, 105 intervention, 107 control (waitlist & self-help book). Female: 130. One or 0 parents Dutch 66. | Treatment satisfaction via CSQ-8, free-text questions on satisfaction and program experiences. | Mean score for eight CSQ-8 items 2.78 (SD 0.58-0.81). | Positive | 2 | 10 |
| Pham et al. (2016) [120] | Anxiety | Mobile app, Flowy | N=63, 31 intervention (free 4-week access), 32 waitlist. Female: 31. Asian, Hispanic, Mixed: 11. | 21 items (1-5); open-ended user feedback and experiences. | Agree/strongly agree: useful 100%, would recommend 89%, easy/simple 92%, fun 83%. | Positive | 2 | 8 |
| Robinson et al. (2010) [121] | GAD | Web-based program, Worry Program, CBT | N=145, 50 technician-assisted, 47 clinician-assisted, and 48 waitlist. Female: 99. | Baseline CEQ; treatment satisfaction with adapted CEQ at post-treatment and three-month follow-up. | 87% very/mostly satisfied, 13% neutral/somewhat dissatisfied, 0% very dissatisfied. Mean confidence in treatment 8, would recommend 9. | Positive | 2 | 7 |
| Ruwaard et al. (2010) [122] | Panic symptoms | Web-based program, CBT | N=58, 27 web-based CBT, 31 waitlist. Female: 42. | Satisfaction ratings on various items (10-point scale). | Posttreatment satisfaction 8.6 (SD 1.3). Follow-up: would recommend 8.9 (SD 1.5). | Positive | 2 | 4 |
| A. J. Schneider et al. (2005) [123] | Phobias and PDs | Web-based program, FearFighter and Managing Anxiety, CBT | N=68, 45 FearFighter, 23 Managing Anxiety (both web-based programs with and without exposure therapy). | Satisfaction at week 10 (after session 6), self-rating and by blinded assessor (0-8) | Self-rated FearFighter 5.5 ± 2.4, Managing Anxiety 4.6 ± 2.1. Assessor-rated FearFighter 6.2 ± 1.6, Managing Anxiety 4.9 ± 2.1. | Positive | 2 | 5 |
| Schrӧder et al. (2017) [124] | Phobias and PDs | Web-based program, ConfID, CBT | N=179, 89 care-as-usual + ConfID, 90 care-as-usual only. Female: 129. | Adapted CSQ-8. Attitudes towards Psychological Online Interventions Questionnaire. | Moderate satisfaction. Retrospective appraisals 49.1%-73.6%. | Positive | 2 | 7 |
| Stech et al. (2016) [125] | PD with or without agoraphobia | Web-based program, THISWAYUP, CBT | N=69, 34 iCBT, 35 iET^m^. Female: 63. Country of birth other than Australia: 8. | Baseline confidence, motivation, two CEQ questions. Overall satisfaction and confidence recommending. | 91% of iCBT and 77 % of iET mostly or very satisfied, 97% and 90% of these groups confident recommending. | Positive | 2 | 10 |
| Titov, Andrews, Schwencke, Drobny, Einstein (2008) (Shyness 1) [126] | SAD | Web-based program, Shyness, CBT | N=99, 50 treatment, 49 waitlist. Female: 58. | At post-treatment, treatment satisfaction questions adapted from CEQ. | All very or mostly satisfied. Mean confidence recommending 9/10. | Positive | 2 | 7 |
| Titov, Andrews, & Schwencke (2008) (Shyness 2) [127] | SAD | Web-based program, Shyness, CBT | N=81, 41 treatment, 40 waitlist. Female: 51. | At post-treatment, treatment satisfaction questions adapted from CEQ. | All very or mostly satisfied. Mean confidence recommending 9/10. | Positive | 2 | 7 |
| Titov, Andrews, Choi, et al. (2008) (Shyness 3) [128] | SAD | Web-based program, Shyness, CBT | N=95, 31 therapist-assisted computerized CBT, 30 self-guided computerized CBT, 34 waitlist. Female: 58. | At post-treatment, treatment satisfaction questions adapted from CEQ. | 97% of therapist-assisted and 62% of self-guided groups very or mostly satisfied. Mean confidence recommending 9/10. | Positive | 2 | 7 |
| Titov, Andrews, Johnston, et al. (2009) [129] | SAD | Web-based program, Shyness, CBT | see Shyness 1 and 2, treatment group participants | At 6 month follow-up, questionnaires on acceptability based on CEQ. | At post-treatment, confidence recommending 9/10. At 6 month follow-up, 8/10. | Positive | 2 | 7 |
| Titov, Andrews, Choi, et al. (2009) (Shyness 4) [130] | SAD | Web-based program, Shyness, CBT | N=163, 81 computerized CBT + telephone reminders, 82 computerized CBT. Female: 85. | At post-treatment, treatment satisfaction questions based on CEQ. | Treatment satisfaction was high but not higher in the computerized CBT + telephone support group. | Positive | 2 | 6 |
| Titov, Andrews, Schwencke, et al. (2009) (Shyness 6) [131] | SAD | Web-based program, Shyness, CBT | N=82, 43 computerized CBT + telephone calls, 39 computerized CBT + forum. Female: 46. | At pre-treatment, CEQ expectancy factors. At post-treatment, treatment satisfaction questions based on CEQ. | Treatment satisfaction was high and similar to results in previous Shyness studies. | Positive | 2 | 9 |
| Titov, Andrews, Robinson, et al. (2009) [132] | GAD | Web-based program, Worry Program, CBT | N=45, 24 treatment (iCBT), 21 waitlist. Female: 34. | At pre-treatment, expectancy factors from CEQ. At post-treatment, treatment satisfaction questions based on CEQ. | 85% very/mostly satisfied, 15% neutral/somewhat satisfied. Mean confidence recommending 9/10. | Positive | 2 | 9 |
| Titov, Andrews, Johnston, et al. (2010) [133] | GAD, SAD, and/or PD | Web-based program, Anxiety Program, CBT | N=78, 40 treatment (immediate treatment & iCBT), 38 control (waitlist/delayed treatment). Female: 53. | At post-treatment, treatment satisfaction questions based on CEQ. | Treatment 71% very/mostly satisfied, 29% neutral/somewhat satisfied, 0 dissatisfied. Mean confidence recommending 8/10. | Positive | 2 | 8 |
| Titov, Andrews, Schwencke, et al. (2010) (Shyness 7) [134] | SAD | Web-based program, Shyness, CBT | N=108, 55 iCBT, 53 iCBT + motivational enhancement strategies. Female: 51. | At pre-treatment, CEQ expectancy factors. At post-treatment, treatment satisfaction questions based on CEQ. | 91% very/mostly satisfied, 10% neutral/somewhat dissatisfied, 0% very dissatisfied. Mean confidence recommending 9/10. | Positive | 2 | 9 |
| Tulbure et al. (2015) [135] | SAD | Web-based program, Internet Social Phobia iSOFIE, CBT | N=76, 38 guided iCBT (iSOFIE), 38 waitlist. Female: 31. | Treatment satisfaction questions | Satisfied 40% or very satisfied 46%, 14% neutral, 0% dissatisfied. Demanding 72% but helpful 100%. | Positive | 2 | 4 |

^a^PD: panic disorder

^b^CBT: cognitive behavioural therapy

^c^SAD: social anxiety disorder

^d^GAD: generalized anxiety disorder

^e^CSQ: Client Satisfaction Questionnaire

^f^ZUF: Fragebogen zur Messung der Patientenzufriedenheit, German version of CSQ

gCBM: cognitive bias modification

^h^iCBT: internet-based cognitive behavioural therapy

^i^TCS: Treatment Credibility Scale

^j^SUS: System Usability Scale

^k^CEQ: Credibility/Expectancy Questionnaire

^l^TSQ: Treatment Satisfaction Questionnaire

^m^iET: internet-delivered exposure therapy

**Table S5.** Description of acceptability results in the 22 non-RCT quantitative studies on digital mental health interventions for anxiety.

| Author (Year) | Diagnosis | Intervention technology, program, treatment paradigm | Sample | Acceptability outcome | Acceptability results | Summarized acceptability result | LOE | Acceptability score |
| --- | --- | --- | --- | --- | --- | --- | --- | --- |
| Aydos et al. (2009) (Shyness 5) [136] | SAD^a^ | Web-based program, Shyness, CBT^b^ | N=17, all intervention. Female: 12. | Treatment satisfaction questionnaire based on CEQ^c^, confidence in treatment, recommending to friend. | 100% very or mostly satisfied. Mean confidence in treatment 9, mean confidence recommending 9. | Positive | 3 | 5 |
| Botella et al. (2009) [137] | SAD (fear of public speaking) | Web-based program, Talk to Me, CBT | N=52, 30 internet-based treatment, 22 face-to-face treatment with therapist. | Motivation and confidence questions (created ad-hoc), satisfaction questions adapted from CEQ. | Satisfaction: items other than aversiveness at post-test 6.96-8.00 (SD 1.43-2.03), follow-up 5.70-7.65 (SD 1.63-2.84). | Positive | 3 | 9 |
| Botella et al. (2007) [138] | SAD (fear of public speaking) | Web-based program, Talk to Me, CBT | N=12, all treatment program. Female: 12. | Motivation, satisfaction, confidence, and treatment utility questions (created ad-hoc) | Mean satisfaction 7.8 (SD 1.69), utility 7.8 (SD 1.93). | Positive | 3 | 6 |
| Botella et al. (2004) [139] | SAD (fear of public speaking) | Web-based program, Talk to Me, CBT | N=1. Female: 1. Other than white: 0. | Motivation, satisfaction, confidence, and treatment utility questions (created ad-hoc), | Motivated at baseline, high confidence. Satisfaction 10 at post-treatment and 8 at follow-up, utility 10 and 8. | Positive | 4 | 8 |
| Carlbring, Furmark, et al. (2006) [140] | SAD | Web-based program, CBT | N=26, all internet-based bibliotherapy self-help program. Female: 18. | Questionnaire on program with Likert-type and open-ended questions | Very or mostly satisfied overall (n=25), neutral (n=1). | Positive | 3 | 7 |
| Draper & Rees (2008) [141] | GAD^d^ | Web-based program, What? Me Worry!?!, CBT | N=3, all received treatment. Female: 1. | Questions on effectiveness, recommending treatment to others with GAD. | Perceived effectiveness 8, 4, and 6. All would recommend. | Mixed | 4 | 6 |
| Ebenfeld et al. (2020) [142] | PD^e^ with or without agoraphobia | Mobile app, GET.ON Panic, CBT | N=10, all GET.ON Panic. Female: 5. Asian or Not specified: 3. | SUS^f^_;_ TAM^g^ subscales; user satisfaction per CSQ^h^ or ZUF^i^. | Mean SUS 84.06 (SD 13.36). Mean satisfaction 30.13/32 (SD 1.64). | Positive | 3 | 8 |
| Gruber et al. (2001) [143] | SAD | Computer-based program, CBT | N=54, 18 12-session cognitive behavioural group treatment, 18 8-session treatment with homework via hand-held computer, 18 waitlist. Female: 28. | Question asking if they believe they would have improved equally without handheld computer, usefulness, awkwardness using handheld computer in public. | Those who felt the computer was useful reported it significantly enhanced their coping, *F*_2, 12_= 4.85, *P*<.03, vs. those who found it not useful. | Not enough information | 3 | 6 |
| Kenardy et al. (2003) [144] | PD | Computer-based program, CBT | N=163, 41 waitlist, 39 CBT6 (6 therapist sessions), 41 CBT6-CA (6 sessions plus computer), 42 CBT12 (12 sessions). Female: 123. | At session one, scale on outcome expectancy (1-5). CBT6-CA condition, satisfaction question (1-5). | No significant differences for outcome expectancy between groups. | Not enough information | 3 | 3 |
| Kenwright et al. (2004) [145] | Phobia or PD | Web-based program, FearFighter, CBT | N=10 internet FearFighter. Female: 4. | Satisfaction questions | Generally satisfied and comfortable. Three preferred face-to-face to internet-guided self-help. | Mixed | 3 | 2 |
| Klein et al. (2008) [146] | PD with or without agoraphobia | Web-based program, Panic Online, CBT | N=6, all Panic Online self-guided. Female: 5. | Semi-structured questions on experiences and attitudes; question on whether they would use self-guided internet intervention again. | All enjoyed the program. Most (n=4) reported it relevant and helpful. All would use this modality again. | Positive | 3 | 8 |
| Krafft et al. (2022) [147] | GAD | Mobile app, ACT Daily, ACT^j^ | N=21, all group ACT and access to ACT Daily. Female: 18. Asian, Other: 9.52%. | At posttreatment, satisfaction via Treatment Evaluation Inventory–Short Form; novel satisfaction questions (1-5). | Mean Treatment Evaluation Inventory–Short Form 28.29 (SD 3.00). Mean helpfulness 3.94 (SD 1.09) but group would have been as helpful without app (mean 3.53, SD 1.13). | Mixed | 3 | 7 |
| Lim et al. (2019) [148] | SAD (and those without) | Mobile app, +Connect app, positive psychology | N=9 (those with social anxiety disorder), all +Connect app. Female: 4. Asian Australian/Asian, Multi-Racial, Other: 2. | Satisfaction ratings relating to understanding, enjoyment, and content helpfulness; qualitative interviews. | ≥50% somewhat or very satisfied. Modules helpful or very helpful 25-50%; at least somewhat helpful, 75-100%. | Positive | 3 | 9 |
| Loo Gee et al. (2021) [149] | SAD | Mobile app, ExposureTherapy, ecological momentary intervention and CBT | N=49, 23 app, 26 control (delayed access to app). Female: 40. | Satisfaction after 4 weeks (1-10). | Mean user satisfaction and acceptability ratings (n=2) 5-8. | Positive | 3 | 6 |
| Miller et al. (2021) [150] | GAD | Web-based program, Daylight, CBT | N=21, 5 2-week baseline, 6 4-week baseline, 10 6-week baseline. Female: 20. Other than white: 0. | Satisfaction at post-intervention via qualitative and quantitative questions. Credibility via CEQ in first weekly survey during intervention period. | Mean CEQ 19.30 (SD 4.78). Mean satisfaction 6.20 (SD 1.99), one completely satisfied, most 7, none totally dissatisfied, qualitatively reported enjoyable and effective. | Positive | 3 | 11 |
| Miralles et al. (2020) [151] | PD with agoraphobia | Mobile app, Symptoms platform, CBT | N=1. Female: 0. | Expectation and satisfaction scale for in vivo exposure and location-based tracking component, six items (each 0-10); SUS. | Expectations rated 10, 10 again at post-treatment. Overall satisfaction and usability of 95. | Positive | 4 | 11 |
| Newman et al. (1997) [152] | PD with or without agoraphobia | Palmtop, CBT | N=18, 9 12-session in-person CBT (CBT12), 9 4-session computer-assisted CBT (CBT4-CA). Female: 15. | At baseline, treatment credibility via two-item measure. At posttreatment, three-item treatment satisfaction measure (items 1-5). | CBT12 and CBT4-CA treatments, mean credibility 8 (SD 1.41) and 7.56 (SD 0.38), satisfaction 12.75 (SD 0.75) and 12.75 (SD 0.62). | Positive | 3 | 5 |
| Oser et al. (2019) [153] | Anxiety | Mobile app, Lantern, CBT | N=812, 593 Lantern, 219 care-as-usual. Female: 569. Other than white: 63. | Helpfulness and satisfaction scale (created ad-hoc): 14 items, each 1-7. | 52% general helpfulness ≥5, 49% satisfaction ≥5. 68% likely to recommend. | Positive | 3 | 6 |
| Pier et al. (2008) [154] | PD with or without agoraphobia | Web-based program, Panic Online, CBT | N=65, 34 Panic Online with face-to-face assistance from GP^k^, 31 Panic Online with email assistance from psychologist. Female: 48. | TCS-M^l^: items rated 0-10 at baseline. | Mean credibility 40.80 of 50 (SD 7.88) program with psychologists, 37.75 (SD 7.25) program with GPs. | Positive | 3 | 8 |
| Shandley et al. (2008) [155] | PD with or without agoraphobia | Web-based program, Panic Online, CBT | N=96, 43 Panic Online + Psychologist, 53 Panic Online + GP. Female: 76. | TCS-M. | No significant differences between groups (*t*_82_=1.96, *P*=.05). | Not enough information | 3 | 6 |
| Tolin et al. (2017) [156] | PD | Respiratory intervention, Freespira, Biofeedback | N=69, all Freespira. Female: 41. African American or Hispanic: 17. | Satisfaction on recommending treatment to friend or family member (0-4). | Post-treatment mean 3.50 (SD 0.77), 2-month follow-up 3.53 (SD 0.73), 12 months 3.33 (SD 0.82). | Positive | 3 | 6 |
| Williams et al. (2014) [157] | SAD | Web-based program, Shyness, CBT | Study 1: N=368, all Shyness Program and primary care in community. Female: 184. Study 2: N=192, primary care referral to university hospital clinic for supervised Shyness Program. Female: 96. | Program satisfaction via two questions, overall satisfaction (1-5) and confidence recommending to friend with social anxiety (1 -10). | Study 1: Mean satisfaction 3.86 (range 1-5; SD .94), confidence recommending 8.09 (SD 2.03). Study 2: mean satisfaction 3.99 (range 1-5; SD .75), confidence recommending 8.35 (SD 1.79). | Positive | 3 | 6 |

^a^SAD: social anxiety disorder

^b^CBT: cognitive behavioural therapy

^c^CEQ: Credibility/Expectancy Questionnaire

^d^GAD: generalized anxiety disorder

^e^PD: panic disorder

^f^SUS: System Usability Scale

^g^TAM: Technology Acceptance Model

^h^CSQ: Client Satisfaction Questionnaire

^i^ZUF: Fragebogen zur Messung der Patientenzufriedenheit, German version of CSQ

^j^ACT: acceptance and commitment therapy

^k^GP: general practitioner

^l^TCS: Treatment Credibility Scale

**Table S6.** Description of acceptability results in the six RCTs on digital mental health interventions for depression and anxiety together.

| Author (Year) | Intervention technology, program, treatment paradigm | Sample | Acceptability outcome | Acceptability results | Summarized acceptability result | LOE | Acceptability score |
| --- | --- | --- | --- | --- | --- | --- | --- |
| Batterham et al. (2021) [158] | Mobile app, web-based program, myCompass, CBT^a^ | N=849, 280 engagement interventions + myCompass, 285 myCompass only, 284 attention control. Female: 646. Non-English language spoken: 29. | Unified Theory of Acceptance and Use of Technology, selected scales. | Mean acceptability 13.36-14.61 (SD 3.02-3.88), performance expectancy 13.36-13.66 (SD 2.68-3.88), effort expectancy 10.24-10.62 (SD 2.1-2.29), across pre-test, post-test, and 6-month follow-up. | Positive | 2 | 14 |
| Kleiboer et al. (2015) [159] | Web-based program, Allesondercontrole, PST^b^ | N=537, 107 internet PST with no support, 324 different supports, 106 waitlist (online info). Female: 348. Non western birthplace: 20. | Global satisfaction with treatment via CSQ-8^c^, 8 items, each on 4-point scale. | Mean CSQ-8 20.8 of 32 (SD 5.1) PST with weekly support, non-specific support 20.0 (SD 5.4), support on request 19.0 (SD 4.7), no support 18.4 (SD 4.9). | Positive | 2 | 8 |
| Newby et al. (2013) [160] | Web-based program, Worry and Sadness Program, CBT | Study 1: N=99, 46 treatment, 53 control. Female: 77. Study 2: N=136, all treatment. Female: 88. | Post-treatment satisfaction ratings on three items, each 1-10. | Mean total satisfaction 25.64 of 30 (SD 3.58) for Study 1, 27.30 (SD 5.69) for Study 2. | Positive | 2 | 6 |
| Proudfoot et al. (2013) [161] | Mobile app and web-based program, myCompass, CBT | N=720, 242 myCompass, 248 attention control, 230 waitlist. Female: 491. | Satisfaction at post-intervention via study-specific measure of perceived usability, content, flexibility and functionality. | Mean satisfaction for program 3.86 (SD 0.82). 83% would recommend, 87.4% would happily use program again. | Positive | 2 | 4 |
| Titov et al. (2013) [162] | Web-based program, Wellbeing Program, CBT | N=257, 100 treatment plus automated email group, 106 treatment group, 51 control (waitlist-deferred treatment control group). Female: 189. | Questions at post-treatment including whether the treatment had been worth their time and whether they would recommend it to a friend with anxiety or depression. | At post-treatment, 95.9% treatment & automated email and 95% treatment only found it worth their time, and 97.3% and 93.7% would recommend. | Positive | 2 | 6 |
| Titov et al. (2011) [163] | Web-based program, Wellbeing Program, CBT | N=74, 37 treatment, 37 waitlist. Female: 54. | At post treatment, satisfaction questions based on CEQ^d^. Questions on acceptability of transdiagnostic treatment protocol to treatment group at 3-month follow-up. | 93% very or mostly satisfied, 7% neutral/somewhat satisfied, 0% dissatisfied. No differences between those with principal diagnosis of depression vs. anxiety, *P*= .43-.70. 100% found it worth their time. 97% would recommend. | Positive | 2 | 9 |

^a^CBT: cognitive behavioural therapy

^b^PST: problem-solving therapy

^c^CSQ: Client Satisfaction Questionnaire

^d^CEQ: Credibility/Expectancy Questionnaire

**Table S7.** Description of acceptability results in the five non-RCT quantitative studies on digital mental health interventions for depression and anxiety.

| Author (Year) | Intervention technology, program, treatment paradigm | Sample | Acceptability outcome | Acceptability results | Summarized acceptability result | LOE | Acceptability score |
| --- | --- | --- | --- | --- | --- | --- | --- |
| Adam et al. (2020) [164] | Mobile app, Rose, mood tracking | N=45, intervention 30, waitlist 15. Female: 29. Black, Hispanic, or Mixed: 9. | Modified Mobile Application Rating Scale on app quality and perceived usability; questions on usability (5-point Likert) | 67% would recommend. Perceived usability 54-83% positive. | Positive | 3 | 5 |
| Johnston et al. (2014) [165] | Web-based program, Mood Mechanic, CBT^a^ | N=18, all treatment group. Female: 14. | Treatment acceptability via 4 questions at post-treatment: two on five-point Likert scale, two with yes/no responses. | 85% very or mostly satisfied course, 15% neutral. 100% found it worth their time and would recommend. | Positive | 3 | 7 |
| Kladnitski et al. (2018) [166] | Web-based program, Mindfulness-Enhanced iCBT MEiCBT, mindfulness | N=22, all MEiCBT. Female: 20. Born in Australia: 20. | Post-treatment program satisfaction (0-5), logic, success teaching relevant skills (1-10). | 69% very satisfied or 25% mostly satisfied, one neutral. | Positive | 3 | 8 |
| Staples et al. (2016) [167] | Web-based program, Wellbeing Plus Course, CBT | N=949, 516 clinic group, 433 research group. Female: 580. | Treatment satisfaction and acceptability at post-treatment via two questions: confidence recommending to a friend and whether it was worth the time (yes/no responses). | 97.3% of clinic group and 93.5% of research group would recommend. 98.7% and 94.3% of these groups found it worth their time. | Positive | 2 | 6 |
| Wright et al. (2002) [168] | Computer-based program, Cognitive Therapy: A Multimedia Learning Program, CT^b^ | N=96, all computer program. Female: 65. | Patient satisfaction via three questions, at program midpoint and end, each 1 to 5, summed for affinity score out of 15. | At midpoint and post, total affinity score 12.3 ± 1.6 and 13.2 ± 1.6 respectively. | Positive | 3 | 7 |

^a^CBT: cognitive behavioural therapy

^b^CT: cognitive therapy

## References

1. Berger T, Hämmerli K, Gubser N, Andersson G, Caspar F. Internet-Based Treatment of Depression: A Randomized Controlled Trial Comparing Guided with Unguided Self-Help. Cognitive Behaviour Therapy. 2011;40(4):251-266. doi:10.1080/16506073.2011.616531
2. Birney AJ, Gunn R, Russell JK, Ary DV. MoodHacker Mobile Web App With Email for Adults to Self-Manage Mild-to-Moderate Depression: Randomized Controlled Trial. JMIR mHealth uHealth. 2016;4(1):e8. doi:10.2196/mhealth.4231
3. Blackwell SE, Browning M, Mathews A, et al. Positive Imagery-Based Cognitive Bias Modification as a Web-Based Treatment Tool for Depressed Adults: A Randomized Controlled Trial. Clinical Psychological Science. 2015;3(1):91-111. doi:10.1177/2167702614560746
4. Bücker L, Schnakenberg P, Karyotaki E, Moritz S, Westermann S. Diminishing Effects After Recurrent Use of Self-Guided Internet-Based Interventions in Depression: Randomized Controlled Trial. J Med Internet Res. 2019;21(10):e14240. doi:10.2196/14240
5. Buntrock C, Ebert D, Lehr D, et al. Effectiveness of a Web-Based Cognitive Behavioural Intervention for Subthreshold Depression: Pragmatic Randomised Controlled Trial. Psychother Psychosom. 2015;84(6):348-358. doi:10.1159/000438673
6. Burton C, Szentagotai Tatar A, McKinstry B, et al. Pilot randomised controlled trial of Help4Mood, an embodied virtual agent-based system to support treatment of depression. J Telemed Telecare. 2016;22(6):348-355. doi:10.1177/1357633X15609793
7. De Graaf LE, Huibers MJH, Riper H, Gerhards SAH, Arntz A. Use and acceptability of unsupported online computerized cognitive behavioral therapy for depression and associations with clinical outcome. Journal of Affective Disorders. 2009;116(3):227-231. doi:10.1016/j.jad.2008.12.009
8. Geraedts AS, Kleiboer AM, Wiezer NM, Cuijpers P, Van Mechelen W, Anema JR. Feasibility of a worker-directed web-based intervention for employees with depressive symptoms. Internet Interventions. 2014;1(3):132-140. doi:10.1016/j.invent.2014.07.001
9. Gómez Penedo JM, Babl AM, Grosse Holtforth M, et al. The Association of Therapeutic Alliance With Long-Term Outcome in a Guided Internet Intervention for Depression: Secondary Analysis From a Randomized Control Trial. J Med Internet Res. 2020;22(3):e15824. doi:10.2196/15824
10. Høifødt RS, Lillevoll KR, Griffiths KM, et al. The Clinical Effectiveness of Web-Based Cognitive Behavioral Therapy With Face-to-Face Therapist Support for Depressed Primary Care Patients: Randomized Controlled Trial. J Med Internet Res. 2013;15(8):e153. doi:10.2196/jmir.2714
11. Johansson O, Bjärehed J, Andersson G, Carlbring P, Lundh LG. Effectiveness of guided internet-delivered cognitive behavior therapy for depression in routine psychiatry: A randomized controlled trial. Internet Interventions. 2019;17:100247. doi:10.1016/j.invent.2019.100247
12. Kelders SM, Bohlmeijer ET, Pots WTM, Van Gemert-Pijnen JEWC. Comparing human and automated support for depression: Fractional factorial randomized controlled trial. Behaviour Research and Therapy. 2015;72:72-80. doi:10.1016/j.brat.2015.06.014
13. Kenter RMF, Cuijpers P, Beekman A, Van Straten A. Effectiveness of a Web-Based Guided Self-help Intervention for Outpatients With a Depressive Disorder: Short-term Results From a Randomized Controlled Trial. J Med Internet Res. 2016;18(3):e80. doi:10.2196/jmir.4861
14. Kok G, Bockting C, Burger H, Smit F, Riper H. Mobile Cognitive Therapy: Adherence and acceptability of an online intervention in remitted recurrently depressed patients. Internet Interventions. 2014;1(2):65-73. doi:10.1016/j.invent.2014.05.002
15. Lappalainen P, Langrial S, Oinas-Kukkonen H, Tolvanen A, Lappalainen R. Web-Based Acceptance and Commitment Therapy for Depressive Symptoms With Minimal Support: A Randomized Controlled Trial. Behav Modif. 2015;39(6):805-834. doi:10.1177/0145445515598142
16. Levin W, Campbell DR, McGovern KB, et al. A computer-assisted depression intervention in primary care. Psychol Med. 2011;41(7):1373-1383. doi:10.1017/S0033291710001935
17. Löbner M, Pabst A, Stein J, et al. Computerized cognitive behavior therapy for patients with mild to moderately severe depression in primary care: A pragmatic cluster randomized controlled trial (@ktiv). Journal of Affective Disorders. 2018;238:317-326. doi:10.1016/j.jad.2018.06.008
18. Lüdtke T, Pult LK, Schröder J, Moritz S, Bücker L. A randomized controlled trial on a smartphone self-help application (Be Good to Yourself) to reduce depressive symptoms. Psychiatry Research. 2018;269:753-762. doi:10.1016/j.psychres.2018.08.113
19. Lukas CA, Berking M. Blending group-based psychoeducation with a smartphone intervention for the reduction of depressive symptoms: results of a randomized controlled pilot study. Pilot Feasibility Stud. 2021;7(1):57. doi:10.1186/s40814-021-00799-y
20. Lukas CA, Eskofier B, Berking M. A Gamified Smartphone-Based Intervention for Depression: Randomized Controlled Pilot Trial. JMIR Ment Health. 2021;8(7):e16643. doi:10.2196/16643
21. Ly KH, Trüschel A, Jarl L, et al. Behavioural activation versus mindfulness-based guided self-help treatment administered through a smartphone application: a randomised controlled trial. BMJ Open. 2014;4(1):e003440. doi:10.1136/bmjopen-2013-003440
22. Ly KH, Topooco N, Cederlund H, et al. Smartphone-Supported versus Full Behavioural Activation for Depression: A Randomised Controlled Trial. Bockting CLH, ed. PLoS ONE. 2015;10(5):e0126559. doi:10.1371/journal.pone.0126559
23. Meyer B, Berger T, Caspar F, Beevers CG, Andersson G, Weiss M. Effectiveness of a Novel Integrative Online Treatment for Depression (Deprexis): Randomized Controlled Trial. J Med Internet Res. 2009;11(2):e15. doi:10.2196/jmir.1151
24. Meyer B, Bierbrodt J, Schröder J, et al. Effects of an Internet intervention (Deprexis) on severe depression symptoms: Randomized controlled trial. Internet Interventions. 2015;2(1):48-59. doi:10.1016/j.invent.2014.12.003
25. Moritz S, Schilling L, Hauschildt M, Schröder J, Treszl A. A randomized controlled trial of internet-based therapy in depression. Behaviour Research and Therapy. 2012;50(7-8):513-521. doi:10.1016/j.brat.2012.04.006
26. Motter JN, Grinberg A, Lieberman DH, Iqnaibi WB, Sneed JR. Computerized cognitive training in young adults with depressive symptoms: Effects on mood, cognition, and everyday functioning. Journal of Affective Disorders. 2019;245:28-37. doi:10.1016/j.jad.2018.10.109
27. Oehler C, Görges F, Rogalla M, Rummel-Kluge C, Hegerl U. Efficacy of a Guided Web-Based Self-Management Intervention for Depression or Dysthymia: Randomized Controlled Trial With a 12-Month Follow-Up Using an Active Control Condition. J Med Internet Res. 2020;22(7):e15361. doi:10.2196/15361
28. Perini S, Titov N, Andrews G. Clinician-Assisted Internet-Based Treatment is Effective for Depression: Randomized Controlled Trial. Aust N Z J Psychiatry. 2009;43(6):571-578. doi:10.1080/00048670902873722
29. Pinto MD, Greenblatt AM, Hickman RL, Rice HM, Thomas TL, Clochesy JM. Assessing the Critical Parameters of eSMART-MH: A Promising Avatar-Based Digital Therapeutic Intervention to Reduce Depressive Symptoms: A Promising Avatar-Based Digital Therapeutic Intervention to Reduce Depressive Symptoms. Perspect Psychiatr Care. 2016;52(3):157-168. doi:10.1111/ppc.12112
30. Reins JA, Boß L, Lehr D, Berking M, Ebert DD. The more I got, the less I need? Efficacy of Internet-based guided self-help compared to online psychoeducation for major depressive disorder. Journal of Affective Disorders. 2019;246:695-705. doi:10.1016/j.jad.2018.12.065
31. Ruwaard J, Schrieken B, Schrijver M, et al. Standardized Web-Based Cognitive Behavioural Therapy of Mild to Moderate Depression: A Randomized Controlled Trial with a Long-Term Follow-Up. Cognitive Behaviour Therapy. 2009;38(4):206-221. doi:10.1080/16506070802408086
32. Smith J, Newby JM, Burston N, et al. Help from home for depression: A randomised controlled trial comparing internet-delivered cognitive behaviour therapy with bibliotherapy for depression. Internet Interventions. 2017;9:25-37. doi:10.1016/j.invent.2017.05.001
33. Stephens VC. University of Exeter; 2014. http://hdl.handle.net/10871/16430
34. Adams S, Penton-Voak IS, Harmer CJ, Holmes EA, Munafò MR. Effects of emotion recognition training on mood among individuals with high levels of depressive symptoms: Study protocol for a randomised controlled trial. Trials. 2013;14:161. doi:10.1186/1745-6215-14-161
35. Titov N, Dear BF, Staples LG, et al. Disorder-specific versus transdiagnostic and clinician-guided versus self-guided treatment for major depressive disorder and comorbid anxiety disorders: A randomized controlled trial. Journal of Anxiety Disorders. 2015;35:88-102. doi:10.1016/j.janxdis.2015.08.002
36. Titov N, Andrews G, Davies M, McIntyre K, Robinson E, Solley K. Internet Treatment for Depression: A Randomized Controlled Trial Comparing Clinician vs. Technician Assistance. García AV, ed. PLoS ONE. 2010;5(6):e10939. doi:10.1371/journal.pone.0010939
37. Tønning ML, Faurholt-Jepsen M, Frost M, et al. The effect of smartphone-based monitoring and treatment on the rate and duration of psychiatric readmission in patients with unipolar depressive disorder: The RADMIS randomized controlled trial. Journal of Affective Disorders. 2021;282:354-363. doi:10.1016/j.jad.2020.12.141
38. Wahbeh H. Internet Mindfulness Meditation Intervention (IMMI) Improves Depression Symptoms in Older Adults. Medicines. 2018;5(4):119. doi:10.3390/medicines5040119
39. Watts S, Mackenzie A, Thomas C, et al. CBT for depression: a pilot RCT comparing mobile phone vs. computer. BMC Psychiatry. 2013;13(1):49. doi:10.1186/1471-244X-13-49
40. Williams AD, Blackwell SE, Mackenzie A, Holmes EA, Andrews G. Combining imagination and reason in the treatment of depression: A randomized controlled trial of internet-based cognitive-bias modification and internet-CBT for depression. Journal of Consulting and Clinical Psychology. 2013;81(5):793-799. doi:10.1037/a0033247
41. Williams AD, O’Moore K, Blackwell SE, Smith J, Holmes EA, Andrews G. Positive imagery cognitive bias modification (CBM) and internet-based cognitive behavioral therapy (iCBT): A randomized controlled trial. Journal of Affective Disorders. 2015;178:131-141. doi:10.1016/j.jad.2015.02.026
42. Wong VWH, Ho FYY, Shi NK, et al. Smartphone-delivered multicomponent lifestyle medicine intervention for depressive symptoms: A randomized controlled trial. Journal of Consulting and Clinical Psychology. 2021;89(12):970-984. doi:10.1037/ccp0000695
43. Addington EL, Cheung EO, Bassett SM, et al. The MARIGOLD study: Feasibility and enhancement of an online intervention to improve emotion regulation in people with elevated depressive symptoms. Journal of Affective Disorders. 2019;257:352-364. doi:10.1016/j.jad.2019.07.049
44. Berman MI, Buckey JC, Hull JG, et al. Feasibility Study of an Interactive Multimedia Electronic Problem Solving Treatment Program for Depression: A Preliminary Uncontrolled Trial. Behavior Therapy. 2014;45(3):358-375. doi:10.1016/j.beth.2014.02.001
45. Burns MN, Begale M, Duffecy J, et al. Harnessing Context Sensing to Develop a Mobile Intervention for Depression. J Med Internet Res. 2011;13(3):e55. doi:10.2196/jmir.1838
46. Callan JA, Dunbar Jacob J, Siegle GJ, et al. CBT MobileWork©: User-Centered Development and Testing of a Mobile Mental Health Application for Depression. Cogn Ther Res. 2021;45(2):287-302. doi:10.1007/s10608-020-10159-4
47. Caplan S, Sosa Lovera A, Reyna Liberato P. A feasibility study of a mental health mobile app in the Dominican Republic: The untold story. International Journal of Mental Health. 2018;47(4):311-345. doi:10.1080/00207411.2018.1553486
48. Cartreine JA, Locke SE, Buckey JC, Sandoval L, Hegel MT. Electronic Problem-Solving Treatment: Description and Pilot Study of an Interactive Media Treatment for Depression. JMIR Res Protoc. 2012;1(2):e11. doi:10.2196/resprot.1925
49. Coutinho E, Alshukri A, De Berardinis J, Dowrick C. POLYHYMNIA Mood – Empowering people to cope with depression through music listening. In: Adjunct Proceedings of the 2021 ACM International Joint Conference on Pervasive and Ubiquitous Computing and Proceedings of the 2021 ACM International Symposium on Wearable Computers. ACM; 2021:188-193. doi:10.1145/3460418.3479334
50. Dahne J, Kustanowitz J, Lejuez CW. Development and Preliminary Feasibility Study of a Brief Behavioral Activation Mobile Application (Behavioral Apptivation) to Be Used in Conjunction With Ongoing Therapy. Cognitive and Behavioral Practice. 2018;25(1):44-56. doi:10.1016/j.cbpra.2017.05.004
51. Dear BF, Zou J, Titov N, et al. Internet-delivered cognitive behavioural therapy for depression: A feasibility open trial for older adults. Aust N Z J Psychiatry. 2013;47(2):169-176. doi:10.1177/0004867412466154
52. Drake G, Csipke E, Wykes T. Assessing your mood online: acceptability and use of Moodscope. Psychol Med. 2013;43(7):1455-1464. doi:10.1017/S0033291712002280
53. Fuller-Tyszkiewicz M, Richardson B, Klein B, et al. A Mobile App–Based Intervention for Depression: End-User and Expert Usability Testing Study. JMIR Ment Health. 2018;5(3):e54. doi:10.2196/mental.9445
54. Gould CE, Carlson C, Ma F, Forman-Hoffman V, Ranta K, Kuhn E. Effects of Mobile App–Based Intervention for Depression in Middle-Aged and Older Adults: Mixed Methods Feasibility Study. JMIR Form Res. 2021;5(6):e25808. doi:10.2196/25808
55. Harper Shehadeh MJ, Abi Ramia J, Cuijpers P, et al. Step-by-Step, an E-Mental Health Intervention for Depression: A Mixed Methods Pilot Study From Lebanon. Front Psychiatry. 2020;10:986. doi:10.3389/fpsyt.2019.00986
56. Jacmon J, Malouff J, Taylor N. Treatment of Major Depression: Effectiveness of Cognitive Behavior Therapy with an Internet Course as a Central Component. EJAP. 2010;5(2):1-8. doi:10.7790/ejap.v5i2.153
57. Kooistra LC, Ruwaard J, Wiersma JE, et al. Development and initial evaluation of blended cognitive behavioural treatment for major depression in routine specialized mental health care. Internet Interventions. 2016;4:61-71. doi:10.1016/j.invent.2016.01.003
58. Mayer G, Hummel S, Oetjen N, et al. User experience and acceptance of patients and healthy adults testing a personalized self-management app for depression: A non-randomized mixed-methods feasibility study. DIGITAL HEALTH. 2022;8:205520762210913. doi:10.1177/20552076221091353
59. Schlosser DA, Campellone TR, Truong B, et al. The feasibility, acceptability, and outcomes of PRIME-D: A novel mobile intervention treatment for depression: S CHLOSSER ET AL . Depress Anxiety. 2017;34(6):546-554. doi:10.1002/da.22624
60. Schuster R, Kalthoff I, Walther A, et al. Effects, Adherence, and Therapists’ Perceptions of Web- and Mobile-Supported Group Therapy for Depression: Mixed-Methods Study. J Med Internet Res. 2019;21(5):e11860. doi:10.2196/11860
61. Stiles-Shields C, Montague E, Kwasny MJ, Mohr DC. Behavioral and cognitive intervention strategies delivered via coached apps for depression: Pilot trial. Psychological Services. 2019;16(2):233-238. doi:10.1037/ser0000261
62. Strauss C, Dunkeld C, Cavanagh K. Is clinician-supported use of a mindfulness smartphone app a feasible treatment for depression? A mixed-methods feasibility study. Internet Interventions. 2021;25:100413. doi:10.1016/j.invent.2021.100413
63. Elliott R, Slatick E, Urman M. Qualitative change process research on psychotherapy: Alternative strategies. Psychologische Beitrage. 2001;43(3):69-111.
64. Tomasino KN, Lattie EG, Ho J, Palac HL, Kaiser SM, Mohr DC. Harnessing Peer Support in an Online Intervention for Older Adults with Depression. The American Journal of Geriatric Psychiatry. 2017;25(10):1109-1119. doi:10.1016/j.jagp.2017.04.015
65. Walsh SM. Development of an Online Intervention Using Positive Psychology for Depression. Queen Mary University of London; 2018. http://qmro.qmul.ac.uk/xmlui/handle/123456789/31871
66. Yeung A, Wang F, Feng F, et al. Outcomes of an online computerized cognitive behavioral treatment program for treating chinese patients with depression: A pilot study. Asian Journal of Psychiatry. 2018;38:102-107. doi:10.1016/j.ajp.2017.11.007
67. Schneider J, Sarrami Foroushani P, Grime P, Thornicroft G. Acceptability of Online Self-Help to People With Depression: Users’ Views of MoodGYM Versus Informational Websites. J Med Internet Res. 2014;16(3):e90. doi:10.2196/jmir.2871
68. Knowles SE, Lovell K, Bower P, Gilbody S, Littlewood E, Lester H. Patient experience of computerised therapy for depression in primary care. BMJ Open. 2015;5(11):e008581. doi:10.1136/bmjopen-2015-008581
69. Ly KH, Janni E, Wrede R, et al. Experiences of a guided smartphone-based behavioral activation therapy for depression: A qualitative study. Internet Interventions. 2015;2(1):60-68. doi:10.1016/j.invent.2014.12.002
70. Allen AR, Newby JM, Mackenzie A, et al. Internet cognitive–behavioural treatment for panic disorder: randomised controlled trial and evidence of effectiveness in primary care. BJPsych open. 2016;2(2):154-162. doi:10.1192/bjpo.bp.115.001826
71. Bell CJ, Colhoun HC, Carter FA, Frampton CM. Effectiveness of computerised cognitive behaviour therapy for anxiety disorders in secondary care. Aust N Z J Psychiatry. 2012;46(7):630-640. doi:10.1177/0004867412437345
72. Berger T, Hohl E, Caspar F. Internet-based treatment for social phobia: a randomized controlled trial. J Clin Psychol. 2009;65(10):1021-1035. doi:10.1002/jclp.20603
73. Berger T, Caspar F, Richardson R, Kneubühler B, Sutter D, Andersson G. Internet-based treatment of social phobia: A randomized controlled trial comparing unguided with two types of guided self-help. Behaviour Research and Therapy. 2011;49(3):158-169. doi:10.1016/j.brat.2010.12.007
74. Berger T, Boettcher J, Caspar F. Internet-based guided self-help for several anxiety disorders: A randomized controlled trial comparing a tailored with a standardized disorder-specific approach. Psychotherapy. 2014;51(2):207-219. doi:10.1037/a0032527
75. Boettcher J, Leek L, Matson L, et al. Internet-Based Attention Bias Modification for Social Anxiety: A Randomised Controlled Comparison of Training towards Negative and Training Towards Positive Cues. Hashimoto K, ed. PLoS ONE. 2013;8(9):e71760. doi:10.1371/journal.pone.0071760
76. Boettcher J, Åström V, Påhlsson D, Schenström O, Andersson G, Carlbring P. Internet-Based Mindfulness Treatment for Anxiety Disorders: A Randomized Controlled Trial. Behavior Therapy. 2014;45(2):241-253. doi:10.1016/j.beth.2013.11.003
77. Boettcher J, Hasselrot J, Sund E, Andersson G, Carlbring P. Combining Attention Training with Internet-Based Cognitive-Behavioural Self-Help for Social Anxiety: A Randomised Controlled Trial. Cognitive Behaviour Therapy. 2014;43(1):34-48. doi:10.1080/16506073.2013.809141
78. Carlbring P, Westling BE, Ljungstrand P, Ekselius L, Andersson G. Treatment of panic disorder via the internet: A randomized trial of a self-help program. Behavior Therapy. 2001;32(4):751-764. doi:10.1016/S0005-7894(01)80019-8
79. Carlbring P, Ekselius L, Andersson G. Treatment of panic disorder via the Internet: a randomized trial of CBT vs. applied relaxation. Journal of Behavior Therapy and Experimental Psychiatry. 2003;34(2):129-140. doi:10.1016/S0005-7916(03)00026-0
80. Carlbring P, Nilsson-Ihrfelt E, Waara J, et al. Treatment of panic disorder: live therapy vs. self-help via the Internet. Behaviour Research and Therapy. 2005;43(10):1321-1333. doi:10.1016/j.brat.2004.10.002
81. Carlbring P, Bohman S, Brunt S, et al. Remote Treatment of Panic Disorder: A Randomized Trial of Internet-Based Cognitive Behavior Therapy Supplemented With Telephone Calls. Am J Psychiatry. Published online 2006.
82. Carter FA, Bell CJ, Colhoun HC. Suitability and acceptability of computerised cognitive behaviour therapy for anxiety disorders in secondary care. Aust N Z J Psychiatry. 2013;47(2):142-152. doi:10.1177/0004867412461384
83. Dear BF, Staples LG, Terides MD, et al. Transdiagnostic versus disorder-specific and clinician-guided versus self-guided internet-delivered treatment for generalized anxiety disorder and comorbid disorders: A randomized controlled trial. Journal of Anxiety Disorders. 2015;36:63-77. doi:10.1016/j.janxdis.2015.09.003
84. Dear BF, Staples LG, Terides MD, et al. Transdiagnostic versus disorder-specific and clinician-guided versus self-guided internet-delivered treatment for Social Anxiety Disorder and comorbid disorders: A randomized controlled trial. Journal of Anxiety Disorders. 2016;42:30-44. doi:10.1016/j.janxdis.2016.05.004
85. Ebenfeld L, Lehr D, Ebert DD, et al. Evaluating a Hybrid Web-Based Training Program for Panic Disorder and Agoraphobia: Randomized Controlled Trial. J Med Internet Res. 2021;23(3):e20829. doi:10.2196/20829
86. Fogliati VJ, Dear BF, Staples LG, et al. Disorder-specific versus transdiagnostic and clinician-guided versus self-guided internet-delivered treatment for panic disorder and comorbid disorders: A randomized controlled trial. Journal of Anxiety Disorders. 2016;39:88-102. doi:10.1016/j.janxdis.2016.03.005
87. Furmark T, Carlbring P, Hedman E, et al. Guided and unguided self-help for social anxiety disorder: randomised controlled trial. Br J Psychiatry. 2009;195(5):440-447. doi:10.1192/bjp.bp.108.060996
88. Gallego MJ, Emmelkamp PMG. The effects of a Dutch version of an Internet- based treatment program for fear of public speaking: A controlled study. 2011;11.
89. Hedman E, Andersson G, Ljótsson B, et al. Internet-Based Cognitive Behavior Therapy vs. Cognitive Behavioral Group Therapy for Social Anxiety Disorder: A Randomized Controlled Non-inferiority Trial. García AV, ed. PLoS ONE. 2011;6(3):e18001. doi:10.1371/journal.pone.0018001
90. Johnston L, Titov N, Andrews G, Spence J, Dear BF. A RCT of a Transdiagnostic Internet-Delivered Treatment for Three Anxiety Disorders: Examination of Support Roles and Disorder-Specific Outcomes. Miles J, ed. PLoS ONE. 2011;6(11):e28079. doi:10.1371/journal.pone.0028079
91. Johnston LJ. Development and Evaluation of a Transdiagnostic Internet-Delivered Cognitive Behavioural Therapy Program for Three Anxiety Disorders. UNSW Sydney; 2012. doi:10.26190/UNSWORKS/15695
92. Jones SL, Hadjistavropoulos HD, Soucy JN. A randomized controlled trial of guided internet-delivered cognitive behaviour therapy for older adults with generalized anxiety. Journal of Anxiety Disorders. 2016;37:1-9. doi:10.1016/j.janxdis.2015.10.006
93. Kiropoulos LA, Klein B, Austin DW, et al. Is internet-based CBT for panic disorder and agoraphobia as effective as face-to-face CBT? Journal of Anxiety Disorders. 2008;22(8):1273-1284. doi:10.1016/j.janxdis.2008.01.008
94. Klein B, Richards JC, Austin DW. Efficacy of internet therapy for panic disorder. Journal of Behavior Therapy and Experimental Psychiatry. 2006;37(3):213-238. doi:10.1016/j.jbtep.2005.07.001
95. Klein B, Austin D, Pier C, et al. Internet‐Based Treatment for Panic Disorder: Does Frequency of Therapist Contact Make a Difference? Cognitive Behaviour Therapy. 2009;38(2):100-113. doi:10.1080/16506070802561132
96. Kok RN, Van Straten A, Beekman ATF, Cuijpers P. Short-Term Effectiveness of Web-Based Guided Self-Help for Phobic Outpatients: Randomized Controlled Trial. J Med Internet Res. 2014;16(9):e226. doi:10.2196/jmir.3429
97. Pham Q, Khatib Y, Stansfeld S, Fox S, Green T. Feasibility and Efficacy of an mHealth Game for Managing Anxiety: “Flowy” Randomized Controlled Pilot Trial and Design Evaluation. Games for Health Journal. 2016;5(1):50-67. doi:10.1089/g4h.2015.0033
98. Robinson E, Titov N, Andrews G, McIntyre K, Schwencke G, Solley K. Internet Treatment for Generalized Anxiety Disorder: A Randomized Controlled Trial Comparing Clinician vs. Technician Assistance. García AV, ed. PLoS ONE. 2010;5(6):e10942. doi:10.1371/journal.pone.0010942
99. Ruwaard J, Broeksteeg J, Schrieken B, Emmelkamp P, Lange A. Web-based therapist-assisted cognitive behavioral treatment of panic symptoms: A randomized controlled trial with a three-year follow-up. Journal of Anxiety Disorders. 2010;24(4):387-396. doi:10.1016/j.janxdis.2010.01.010
100. Schneider AJ, Mataix-Cols D, Marks IM, Bachofen M. Internet-Guided Self-Help with or without Exposure Therapy for Phobic and Panic Disorders. Psychother Psychosom. 2005;74(3):154-164. doi:10.1159/000084000
101. Schröder J, Jelinek L, Moritz S. A randomized controlled trial of a transdiagnostic Internet intervention for individuals with panic and phobias – One size fits all. Journal of Behavior Therapy and Experimental Psychiatry. 2017;54:17-24. doi:10.1016/j.jbtep.2016.05.002
102. Stech EP, Chen AZ, Sharrock MJ, et al. Internet-delivered exposure therapy versus internet-delivered cognitive behavioral therapy for panic disorder: A pilot randomized controlled trial. Journal of Anxiety Disorders. 2021;79:102382. doi:10.1016/j.janxdis.2021.102382
103. Titov N, Andrews G, Schwencke G, Drobny J, Einstein D. Shyness 1: Distance Treatment of Social Phobia Over the Internet. Aust N Z J Psychiatry. 2008;42(7):585-594. doi:10.1080/00048670802119762
104. Titov N, Andrews G, Schwencke G. Shyness 2: Treating Social Phobia Online: Replication and Extension. Aust N Z J Psychiatry. 2008;42(7):595-605. doi:10.1080/00048670802119820
105. Titov N, Andrews G, Choi I, Schwencke G, Mahoney A. Shyness 3: Randomized Controlled Trial of Guided Versus Unguided Internet-Based CBT for Social Phobia. Aust N Z J Psychiatry. 2008;42(12):1030-1040. doi:10.1080/00048670802512107
106. Titov N, Andrews G, Johnston L, Schwencke G, Choi I. Shyness Programme: Longer Term Benefits, Cost-Effectiveness, and Acceptability. Aust N Z J Psychiatry. 2009;43(1):36-44. doi:10.1080/00048670802534424
107. Titov N, Andrews G, Choi I, Schwencke G, Johnston L. Randomized Controlled Trial of Web-Based Treatment of Social Phobia Without Clinician Guidance. Aust N Z J Psychiatry. 2009;43(10):913-919. doi:10.1080/00048670903179160
108. Titov N, Andrews G, Schwencke G, Solley K, Johnston L, Robinson E. An RCT Comparing Effect of Two Types of Support on Severity of Symptoms for People Completing Internet-Based Cognitive Behaviour Therapy for Social Phobia. Aust N Z J Psychiatry. 2009;43(10):920-926. doi:10.1080/00048670903179228
109. Titov N, Andrews G, Robinson E, et al. Clinician-Assisted Internet-Based Treatment is Effective for Generalized Anxiety Disorder: Randomized Controlled Trial. Aust N Z J Psychiatry. 2009;43(10):905-912. doi:10.1080/00048670903179269
110. Titov N, Andrews G, Johnston L, Robinson E, Spence J. Transdiagnostic Internet treatment for anxiety disorders: A randomized controlled trial. Behaviour Research and Therapy. 2010;48(9):890-899. doi:10.1016/j.brat.2010.05.014
111. Titov N, Andrews G, Schwencke G, Robinson E, Peters L, Spence J. Randomized Controlled Trial of Internet Cognitive Behavioural Treatment for Social Phobia with and Without Motivational Enhancement Strategies. Aust N Z J Psychiatry. 2010;44(10):938-945. doi:10.3109/00048674.2010.493859
112. Tulbure BT, Szentagotai A, David O, et al. Internet-Delivered Cognitive-Behavioral Therapy for Social Anxiety Disorder in Romania: A Randomized Controlled Trial. Dichter GS, ed. PLoS ONE. 2015;10(5):e0123997. doi:10.1371/journal.pone.0123997
113. Aydos L, Titov N, Andrews G. Shyness 5: The Clinical Effectiveness of Internet-Based Clinician-Assisted Treatment of Social Phobia. Australas Psychiatry. 2009;17(6):488-492. doi:10.1080/10398560903284943
114. Botella C, Gallego MJ, Garcia-Palacios A, Baños RM, Quero S, Alcañiz M. The acceptability of an Internet-based self-help treatment for fear of public speaking. British Journal of Guidance & Counselling. 2009;37(3):297-311. doi:10.1080/03069880902957023
115. Botella C, Guillen V, Banos RM, García-Palacios A, Gallego MJ, Alcaniz M. Telepsychology and Self-help: The Treatment of Fear of Public Speaking. Cognitive and Behavioral Practice. 2007;14(1):46-57. doi:10.1016/j.cbpra.2006.01.007
116. Botella C, Hofmann SG, Moscovitch DA. A self-applied, Internet-based intervention for fear of public speaking. J Clin Psychol. 2004;60(8):821-830. doi:10.1002/jclp.20040
117. Carlbring P, Furmark T, Steczkó J, Ekselius L, Andersson G. An open study of Internet-based bibliotherapy with minimal therapist contact via email for social phobia. Clinical Psychologist. 2006;10(1):30-38. doi:10.1080/13284200500378662
118. Draper M, Rees CS, Nathan PR. Internet-Based Self-Management of Generalised Anxiety Disorder: A Preliminary Study. Behav change. 2008;25(4):229-244. doi:10.1375/bech.25.4.229
119. Ebenfeld L, Kleine Stegemann S, Lehr D, et al. A mobile application for panic disorder and agoraphobia: Insights from a multi-methods feasibility study. Internet Interventions. 2020;19:100296. doi:10.1016/j.invent.2019.100296
120. Gruber K, Moran PJ, Roth WT, Taylor CB. Computer-assisted cognitive behavioral group therapy for social phobia. Behavior Therapy. 2001;32(1):155-165. doi:10.1016/S0005-7894(01)80050-2
121. Kenardy JA, Dow MGT, Johnston DW, Newman MG, Thomson A, Taylor CB. A Comparison of Delivery Methods of Cognitive-Behavioral Therapy for Panic Disorder: An International Multicenter Trial. Journal of Consulting and Clinical Psychology. 2003;71(6):1068-1075. doi:10.1037/0022-006X.71.6.1068
122. Kenwright M, Marks IM, Gega L, Mataix-Cols D. Computer-aided self-help for phobia/panic via internet at home: A pilot study. Br J Psychiatry. 2004;184(5):448-449. doi:10.1192/bjp.184.5.448
123. Klein B, Shandley K, Austin D, Nordin S. A Pilot Trial of ‘Panic Online’ as a Self-Guided Treatment for Panic Disorder. EJAP. 2008;4(2):25-30. doi:10.7790/ejap.v4i2.136
124. Krafft J, Ong CW, Davis CH, Petersen JM, Levin ME, Twohig MP. An Open Trial of Group Acceptance and Commitment Therapy With an Adjunctive Mobile App for Generalized Anxiety Disorder. Cognitive and Behavioral Practice. 2022;29(4):846-859. doi:10.1016/j.cbpra.2021.05.008
125. Lim MH, Rodebaugh TL, Eres R, Long KM, Penn DL, Gleeson JFM. A Pilot Digital Intervention Targeting Loneliness in Youth Mental Health. Front Psychiatry. 2019;10:604. doi:10.3389/fpsyt.2019.00604
126. Loo Gee B, Batterham PJ, Gulliver A, Reynolds J, Griffiths KM. An Ecological Momentary Intervention for people with social anxiety: A descriptive case study. Informatics for Health and Social Care. 2021;46(4):370-398. doi:10.1080/17538157.2021.1896525
127. Miller CB, Gu J, Henry AL, et al. Feasibility and efficacy of a digital CBT intervention for symptoms of Generalized Anxiety Disorder: A randomized multiple-baseline study. Journal of Behavior Therapy and Experimental Psychiatry. 2021;70:101609. doi:10.1016/j.jbtep.2020.101609
128. Miralles I, Granell C, García-Palacios A, et al. Enhancing In Vivo Exposure in the Treatment of Panic Disorder and Agoraphobia Using Location-Based Technologies: A Case Study. Clinical Case Studies. 2020;19(2):145-159. doi:10.1177/1534650119892900
129. Newman MG, Kenardy J, Herman S, Taylor CB. Comparison of palmtop-computer-assisted brief cognitive-behavioral treatment to cognitive-behavioral treatment for panic disorder. Journal of Consulting and Clinical Psychology. 1997;65(1):178-183. doi:10.1037/0022-006X.65.1.178
130. Oser M, Wallace ML, Solano F, Szigethy EM. Guided Digital Cognitive Behavioral Program for Anxiety in Primary Care: Propensity-Matched Controlled Trial. JMIR Ment Health. 2019;6(4):e11981. doi:10.2196/11981
131. Pier C, Austin DW, Klein B, et al. A controlled trial of internet-based cognitive-behavioural therapy for panic disorder with face-to-face support from a general practitioner or email support from a psychologist. Ment Health Fam Med. 2008;5(1):29-39.
132. Shandley K, Austin DW, Klein B, et al. Therapist-Assisted, Internet-Based Treatment for Panic Disorder: Can General Practitioners Achieve Comparable Patient Outcomes to Psychologists? J Med Internet Res. 2008;10(2):e14. doi:10.2196/jmir.1033
133. Tolin DF, McGrath PB, Hale LR, Weiner DN, Gueorguieva R. A Multisite Benchmarking Trial of Capnometry Guided Respiratory Intervention for Panic Disorder in Naturalistic Treatment Settings. Appl Psychophysiol Biofeedback. 2017;42(1):51-58. doi:10.1007/s10484-017-9354-4
134. Williams AD, O’Moore K, Mason E, Andrews G. The effectiveness of internet cognitive behaviour therapy (iCBT) for social anxiety disorder across two routine practice pathways. Internet Interventions. 2014;1(4):225-229. doi:10.1016/j.invent.2014.11.001
135. Batterham PJ, Calear AL, Sunderland M, et al. A Brief Intervention to Increase Uptake and Adherence of an Internet-Based Program for Depression and Anxiety (Enhancing Engagement With Psychosocial Interventions): Randomized Controlled Trial. J Med Internet Res. 2021;23(7):e23029. doi:10.2196/23029
136. Kleiboer A, Donker T, Seekles W, Van Straten A, Riper H, Cuijpers P. A randomized controlled trial on the role of support in Internet-based problem solving therapy for depression and anxiety. Behaviour Research and Therapy. 2015;72:63-71. doi:10.1016/j.brat.2015.06.013
137. Newby JM, Mackenzie A, Williams AD, et al. Internet cognitive behavioural therapy for mixed anxiety and depression: a randomized controlled trial and evidence of effectiveness in primary care. Psychol Med. 2013;43(12):2635-2648. doi:10.1017/S0033291713000111
138. Proudfoot J, Clarke J, Birch MR, et al. Impact of a mobile phone and web program on symptom and functional outcomes for people with mild-to-moderate depression, anxiety and stress: a randomised controlled trial. BMC Psychiatry. 2013;13(1):312. doi:10.1186/1471-244X-13-312
139. Titov N, Dear BF, Johnston L, et al. Improving Adherence and Clinical Outcomes in Self-Guided Internet Treatment for Anxiety and Depression: Randomised Controlled Trial. Andersson G, ed. PLoS ONE. 2013;8(7):e62873. doi:10.1371/journal.pone.0062873
140. Titov N, Dear BF, Schwencke G, et al. Transdiagnostic internet treatment for anxiety and depression: A randomised controlled trial. Behaviour Research and Therapy. 2011;49(8):441-452. doi:10.1016/j.brat.2011.03.007
141. Adam A, Jain A, Pletnikova A, et al. Use of a Mobile App to Augment Psychotherapy in a Community Psychiatric Clinic: Feasibility and Fidelity Trial. JMIR Form Res. 2020;4(7):e17722. doi:10.2196/17722
142. Johnston L, Dear BF, Gandy M, et al. Exploring the efficacy and acceptability of Internet-delivered cognitive behavioural therapy for young adults with anxiety and depression: An open trial. Aust N Z J Psychiatry. 2014;48(9):819-827. doi:10.1177/0004867414527524
143. Kladnitski N, Smith J, Allen A, Andrews G, Newby JM. Online mindfulness-enhanced cognitive behavioural therapy for anxiety and depression: Outcomes of a pilot trial. Internet Interventions. 2018;13:41-50. doi:10.1016/j.invent.2018.06.003
144. Staples LG, Fogliati VJ, Dear BF, Nielssen O, Titov N. Internet-delivered treatment for older adults with anxiety and depression: implementation of the Wellbeing Plus Course in routine clinical care and comparison with research trial outcomes. BJPsych open. 2016;2(5):307-313. doi:10.1192/bjpo.bp.116.003400
145. Wright JH, Wright AS, Salmon P, et al. Development and Initial Testing of a Multimedia Program for Computer-Assisted Cognitive Therapy. APT. 2002;56(1):76-86. doi:10.1176/appi.psychotherapy.2002.56.1.76
